# Gamified Feedback-Based Training System for Pediatric Asthma Inhaler Use: Mixed Methods Randomized Crossover Study

**DOI:** 10.2196/85673

**Published:** 2026-05-04

**Authors:** Haoyu Zhang, Xiaoying Li

**Affiliations:** 1 School of Industrial Design, Hubei University of Technology, No 28, Nanli Road, Hongshan District, Wuhan, Hubei Province, 430068, China, 86 18907101857

**Keywords:** gamification intervention, human-computer interaction, inhaler training, pediatric asthma, treatment adherence

## Abstract

**Background:**

Asthma is a prevalent chronic respiratory condition among children worldwide. Inhalation therapy is the primary treatment method, but children often make errors in its use and exhibit poor adherence, which impacts treatment effectiveness. Therefore, interventions to improve inhalation techniques and enhance adherence are urgently needed.

**Objective:**

This study aimed to develop and evaluate BreatheBuddy, developed by Haoyu Zhang, a training system incorporating gamified feedback designed to enhance inhalation skills and treatment adherence in children with asthma.

**Methods:**

This study used a single-factor repeated-measures design and recruited 20 children aged 6 to 8 years (10 boys and 10 girls), all of whom had prior experience with inhalers. The experimental group used the BreatheBuddy system, which combines a physical inhaler with an interactive game-based software. The system provides real-time animated feedback based on data from inhalation, breath-holding, and exhalation to guide the rhythm and depth of inhalation. The control group used a conventional inhaler method without a gamified system. Inhalation accuracy, adherence, and satisfaction were assessed using the respiration sensor, the Player Experience of Need Satisfaction scale, the Game User Experience Satisfaction Scale (GUESS), and the System Usability Scale (SUS) scales. Statistical comparisons between the groups were conducted using paired *t* tests and Mann-Whitney *U* tests to analyze differences.

**Results:**

The experimental group demonstrated significant improvements in inhalation accuracy, with longer breath-holding times and more stable breathing patterns compared to the control group (*P*<.001). The experimental group also exhibited significantly higher engagement and motivation, with Player Experience of Need Satisfaction (standardized score=93.83) and GUESS (median 87.92, IQR 86.54-88.46) scores markedly higher than those of the control group. Usability scores for the experimental group were also superior, with an SUS score of 88.96 (*P*<.001). Additionally, children in the experimental group showed reduced anxiety and improved focus during training.

**Conclusions:**

BreatheBuddy effectively optimized children’s inhalation skills, boosted treatment adherence, and relieved inhalation-related anxiety. Different from conventional non-gamified training or simple game-based distraction, this study integrated breathing behaviors into core game interaction. With dynamic respiratory rhythm feedback, the system unifies skill training, motivation promotion, and emotional regulation. Combined with standard inhaler operation and immersive gamified interaction, it presents a novel behavior-oriented design paradigm. This work provides empirical evidence for gamified intervention in pediatric respiratory treatment and offers a practical auxiliary tool for clinical daily training to strengthen children’s self-management. Further research will focus on personalized adjustment and wider clinical application of the system.

## Introduction

Asthma and chronic obstructive pulmonary disease are common chronic respiratory disorders. Among them, asthma has emerged as one of the most prevalent chronic diseases in children worldwide [1-4]. Inhalation therapy, primarily through inhalers, is the preferred treatment modality, as it delivers medication directly to the airways, enhances local drug concentration, and minimizes systemic side effects [5-7]. However, effective asthma control remains a clinical challenge. Studies indicate that approximately 55.1% of pediatric patients with asthma fail to achieve optimal therapeutic outcomes, largely due to poor treatment adherence and improper inhalation technique [8-11]. Surveys reveal that nearly 50% of patients cannot use inhalers correctly, and up to 80% make errors during operation, resulting in excessive oropharyngeal drug deposition, reduced pulmonary deposition [12,13], diminished treatment efficacy, and increased risk of acute exacerbations [14-16].

In pediatric patients, misuse of inhalers is particularly problematic due to physiological characteristics such as narrow airways, small tidal volumes, and high respiratory rates, as well as difficulties in coordination. Observational studies suggest that only 8%‐22% of children can use inhalers correctly [17-21]. Despite guideline recommendations for inhaler education at every clinical visit, about 25% of patients receive no instruction [22,23], and only 10% of children undergo inhalation technique assessment during consultations [24]. This educational gap limits improvements in treatment outcomes. Evidence shows that inhalation training significantly prolongs inspiratory duration and increases drug deposition [25]. Moreover, multidisciplinary reviews and clinical guidelines emphasize the importance of selecting age- and physiology-appropriate devices (eg, masks, spacers, or nebulizers) and reinforcing repeated training [26]. Nevertheless, limited cognitive development in children contributes to poor treatment adherence, with approximately 74.7% of pediatric patients exhibiting nonadherent behaviors—such as crying or refusing to wear masks—which significantly compromise therapeutic effectiveness [27]. Improving adherence in children has thus become a central focus of clinical care.

To enhance the treatment experience, numerous studies have explored various intervention strategies, such as distraction-based interventions, inhaler usage training, and the optimization of the clinical environment [28]. Moreover, educational interventions and inhaler training have been shown to significantly improve patient adherence [29]. Certain hospitals integrate colorful, cartoon-themed designs into the environment to alleviate children’s anxiety [30]. Additionally, health care providers use games, videos, and relaxation techniques to divert children’s attention and reduce their stress levels. In terms of equipment design, more compact and visually engaging nebulizers have been developed, including toy nebulizers and toy masks, which improve the airtight seal and help reduce anxiety [31]. Although these traditional approaches have seen some advancements, they still necessitate considerable resource investment, including the time required for implementing therapeutic games, additional training, institutional support, and the need for further financial resources and infrastructure modifications [32].

It is crucial to highlight that the use of most inhalation devices is based on three key steps: inhalation, breath-holding, and exhalation, which are essential for the effectiveness of inhalation therapy. Research has shown that the depth of inhalation, breath-holding duration, and exhalation rhythm directly influence the pulmonary deposition efficiency of medication [33,34]. Specifically, the duration of breath-holding is strongly correlated with the amount of medication deposited in the lungs. Proper breath-holding duration significantly enhances the deposition ratio in the lungs, thereby improving therapeutic outcomes. This is a critical aspect that should be emphasized in inhaler training for children [35].

In recent years, the use of gamification and digital serious games for the self-management of chronic diseases in children and adolescents has rapidly grown, becoming a major form of digital health intervention. These approaches effectively enhance health knowledge and self-management skills, particularly among children with chronic illnesses, demonstrating positive effects [36]. Systematic reviews show that digital interventions such as smart inhalers, mobile apps, and reminder messages positively impact asthma medication adherence and clinical outcomes [37]. Studies have found that augmented reality educational tools co-designed with children can significantly improve inhaler skills and increase engagement [38]. Training methods combining mobile games have also been shown to improve children’s inhalation techniques and quality of life [39]. Additionally, smart inhalers equipped with audio and visual feedback systems have effectively improved medication adherence in children, and their real-time feedback enhances the interactivity and engagement of treatment [40]. Review papers indicate that electronic monitoring devices play a vital role in helping children use inhalers correctly, effectively improving inhalation techniques [41]. Innovative smart devices and gamification designs have also shown significant improvements in adherence and engagement [12]. Finally, computer interactive narratives and serious games have been found to increase children’s adherence to inhaler use and improve therapeutic outcomes [42]. Although different types of inhalers (including dry powder inhalers, pressurized metered-dose inhalers, and combination devices) have different operational details, they all focus on regulating the breathing rhythm [43] (Multimedia Appendix 1). These studies collectively indicate that smart technologies and gamification designs are effective in enhancing self-management of chronic diseases in children, particularly in improving adherence and therapeutic outcomes. However, existing gamified devices have not sufficiently considered the inhaler itself as an input device. Although self-determination theory and flow theory provide psychological foundations for gamification [44], and frameworks such as Mechanics, Dynamics, and Aesthetics (MDA) offer practical guidance for system design [45], current applications fail to fully integrate children’s physiological characteristics and the specific context of inhalation therapy, which limits their effectiveness. Additionally, many current gamified interventions rely on external devices such as tablets and smartphones, rather than integrating the inhaler itself into the interactive experience [46-48]. This may result in reduced interactivity and misalignment with the treatment process, ultimately limiting the devices’ ability to improve children’s breathing accuracy and medication adherence.

This study introduces BreatheBuddy, developed by Haoyu Zhang, a gamified feedback-based inhaler training system for children with asthma. The purpose of this study is to guide children in performing correct breathing exercises through real-time animations and gamified feedback, thereby enhancing their inhaler technique and treatment adherence. By integrating gamified intervention theory with human-computer interaction technology, the system aims to improve children’s inhaler use behaviors and, ultimately, enhance treatment outcomes.

The main research questions (RQs) of this study are as follows:

- RQ1: How can an interactive inhaler training tool with a gamification system be designed for children?
- RQ2: Does the accuracy of children’s inhalation/breath-holding/exhalation rhythm improve, thereby enhancing therapeutic outcomes?
- RQ3: Does children’s treatment adherence improve?

## Methods

### System Design and Implementation

Before system design, the research team conducted surveys and semistructured interviews with children, parents, and health care professionals. This preliminary study aimed to identify the experiences, challenges, and needs of pediatric patients, caregivers, and experts in the process of using asthma inhalers. The findings served as direct empirical evidence for answering RQ1 (“How can an interactive inhaler training tool with a gamification system be designed for children?”) and laid the foundation for the design of the BreatheBuddy system.

### Multistakeholder Engagement

#### Sample and Participant Recruitment

To explore children’s psychological states and coping strategies during inhaler use, questionnaires were administered to 20 preschool children (ages 5‐8 years), 7 parents (ages 28‐37 years), and 2 pediatric health care professionals (ages 29‐31 years). Eligible children were required to have prior experience with nebulization and at least one use of an inhaler, while children with severe cognitive or language impairments were excluded. The parents were the primary caregivers of the aforementioned children, all of whom had accompanied their children during inhalation therapy. The health care professionals were 2 frontline pediatric nurses with teaching and guidance experience in inhaler use. All participants were recruited from local community health centers and kindergartens. Ethical guidelines were strictly followed, and all data were anonymized during collection and analysis.

#### Questionnaire Structure

The questionnaire was initially drafted by the research team and subsequently reviewed and revised by 2 health care professionals. The final version consisted of 3 sections: child-friendly questions (focusing on children’s emotional experiences, common difficulties, and motivations during inhalation therapy), parent supplementary questions (designed to capture observations of children’s behavioral responses while also exploring parental support strategies and attitudes), and health care professional questions (addressing inhaler operation errors, adherence barriers, and the potential clinical value of gamification). To ensure the validity of the data and maintain participant focus, a cognitive attention validation task was incorporated into the questionnaire. This task is intended to assess whether the participants are fully engaged and to exclude responses from participants who show signs of inattentiveness or inconsistent answers.

#### Interviews

Interviews were conducted either face-to-face or via online video conferencing, lasting 5‐10 minutes each. Two researchers shared responsibilities: 1 facilitated and guided the interview, while the other documented and observed nonverbal behaviors. All interviews were recorded with participant consent, transcribed, anonymized, and subsequently used for qualitative analysis.

### Children’s Responses

Survey results showed that 50% of children reported disliking inhaler use, while 45% felt fearful, often exhibiting resistance or irritability. Many children commonly experience difficulties with inhaler operation, particularly in controlling inhalation timing and depth, frequently resorting to rapid nasal breathing, which results in poor synchronization with the device. Additionally, 90% reported that breath-holding was challenging, further contributing to resistance. Many children also displayed uncooperative behaviors during inhaler use; frightened children often cried, refused treatment, or sought parental help, with few attempts at self-regulation. Some children also perceived the training process as lacking sufficient engagement or playfulness.

### Parents’ Perspectives

Parents were particularly concerned about children’s lack of adherence. About 42.86% of parents reported that their children often resisted or failed to cooperate, which they believed directly compromised treatment efficacy. Common parental coping strategies included enforced inhaler use (71.43%), use of electronic devices as distraction tools (57.14%), and continued use despite negative emotional states (28.57%). These findings suggest that many parents experienced significant stress and sometimes resorted to forceful measures to ensure inhaler use. Nevertheless, all parents reported using verbal encouragement to help children overcome resistance, indicating attempts to foster positive engagement despite the challenges. Around 57.14% of parents were also concerned about children’s technical difficulties during inhaler use, such as maintaining proper breathing rhythm. These issues, intertwined with poor adherence, caused distress among parents and raised concerns about therapeutic effectiveness.

Finally, parents expressed both interest and concerns regarding future gamified inhaler systems. While many were worried about insufficient gaming effects (57.14%) and potential risk of addiction (57.14%), their most critical concern was whether gamification could compromise medication safety and efficacy. These results suggest that parents expect inhaler systems not only to provide enjoyable experiences but also to be designed with therapeutic effectiveness as the foremost priority.

### Opinions of Health Care Professionals

Health care professionals highlighted that common errors made by children when using inhalers include inhaling too rapidly, insufficient breath-holding, and exhaling too early, all of which severely compromise drug deposition. They recommended incorporating stepwise rhythm guidance into the training tool to accommodate differences in lung function across age groups. In addition, concerns were raised that an excessive emphasis on gamification might produce counterproductive effects. Therefore, the degree of playfulness and interactivity should be carefully calibrated to reduce children’s resistance and to avoid monotonous training experiences. Consideration should also be given to the compatibility of the system with clinical workflows.

### System Design Concept and Interaction Mechanism

In the design of the system, we used the MDA game design framework to guide the development of the gamification elements. This framework improves the user experience by refining the key aspects of gameplay—MDA—ensuring the game is both engaging and effective in supporting children’s respiratory training. The software component was developed by the first author under the supervision of their doctoral advisor, using the TouchDesigner platform (developed by Haoyu Zhang). A real-time data feedback mechanism was integrated to enhance the interactivity and responsiveness of the game interface. For the hardware design, the first author was responsible for designing the inhaler, consulting with a structural design expert during the design phase to ensure the device’s functionality and safety. Additionally, the doctoral advisor provided valuable guidance and oversight regarding the feasibility and real-world application of the hardware.

#### Conceptual Design

**Figure 1.**
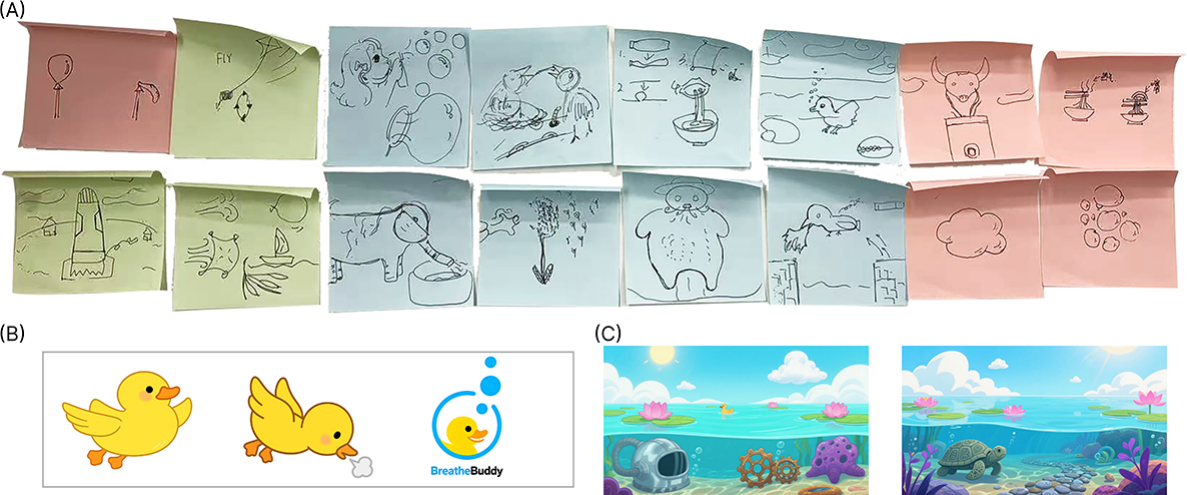
(A) Workshop blueprint design proposals and selections, (B) selection and design of the Little Yellow Duck character, and (C) selection and design of the diving scene.

At the stage of concept development, the research team organized multiple workshop-style discussions at the User Experience and Interaction Design Laboratory of Hubei University of Technology, involving designers, child psychology researchers, and potential users. Through prototype demonstrations and scenario simulations, the team gradually established the narrative framework of “Little Yellow Duck Diving” for respiratory training. The discussions revealed that this narrative not only received positive feedback from both children and parents but was also perceived as effective in alleviating tension and anxiety during the training process. Figure 1A shows the design and selection of the training environment layout, aimed at enhancing the interactive experience; Figure 1B shows the selection and design of the yellow duck character, an engaging and appealing character; and Figure 1C shows the selection and design of the underwater-themed scene, incorporating diving elements to illustrate the various stages of breathing exercise.

Selecting the Little Yellow Duck’s diving adventure as the narrative backdrop was guided by the following design considerations: (1) the Little Yellow Duck is an innocent, cute, and highly approachable character that can effectively capture children’s attention and evoke emotional resonance; (2) within the respiratory-training feedback mechanism, if a child’s breath-hold duration is insufficient or their breathing rhythm deviates from the target pattern, the character will be unable to complete the diving action. Compared with terrestrial animals such as rabbits or cats, a diving scenario may trigger negative associations (eg, “drowning” or “suffocation”). As a natural adapter to aquatic environments, the Little Yellow Duck can plausibly surface even when a dive attempt is unsuccessful, which better aligns with its implied physiological affordances. This helps avoid introducing threatening contexts and supports a safe, friendly training experience; and (3) training for inhalation, breath-holding, and exhalation aligns naturally with the actions involved in diving: inhalation represents preparation before submerging, breath-holding simulates the underwater state, and exhalation corresponds to resurfacing. This close coupling between action and training further strengthens children’s understanding and engagement. The following narrative logic establishes an intuitive mapping to respiratory-training actions: (1) inhalation phase: the child inhales deeply to charge the Little Yellow Duck with energy; (2) breath-holding phase: the Little Yellow Duck holds its breath and dives underwater, preparing to traverse beneath the surface; and (3) exhalation phase: as the child exhales, the Little Yellow Duck steadily resurfaces, completing the challenge.

#### Design of the Game Prototype

This study applied the MDA game design framework to guide the design and development process, aiming to improve the gameplay experience by optimizing three core elements. The mechanics define the fundamental rules and interactions, ensuring operational simplicity; the dynamics provide real-time feedback on children’s breathing performance, dynamically adjusting difficulty to guide proper breathing; and the aesthetics enrich the affective experience through visual and auditory design, thereby enhancing engagement [45].

**Figure 2.**
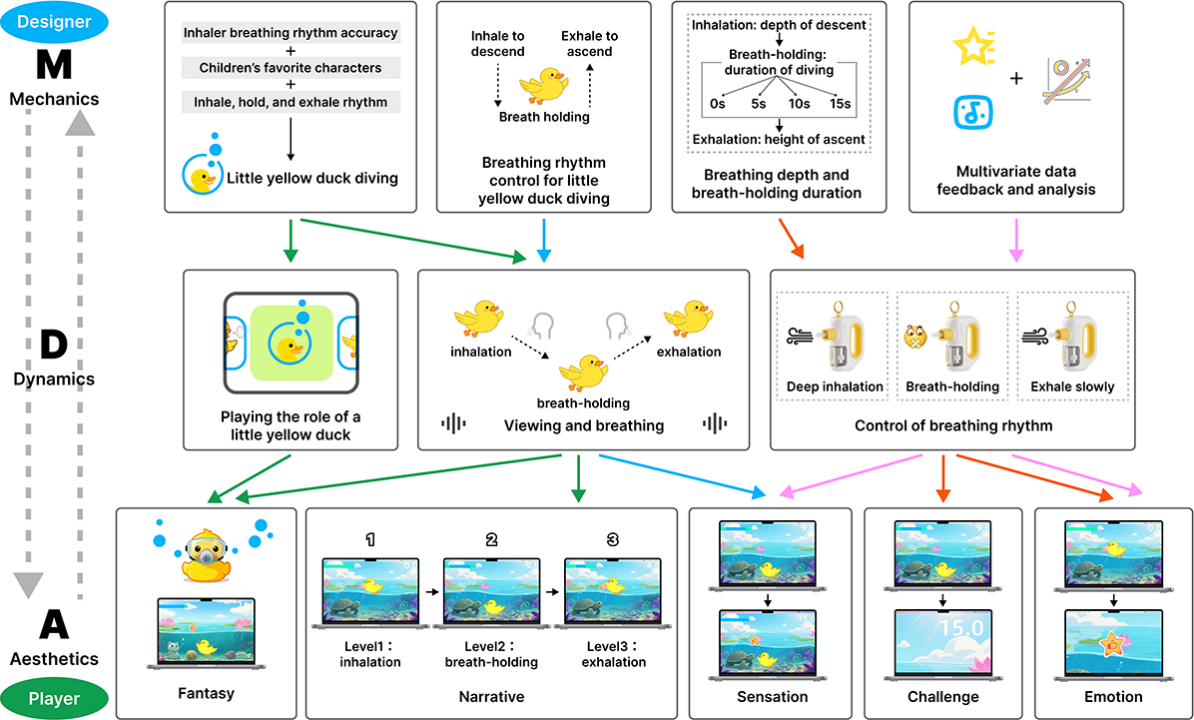
BreatheBuddy system design based on the Mechanics, Dynamics, and Aesthetics (MDA) framework.

From the user perspective, the game is both entertaining and supportive of respiratory training; from the designer perspective, the MDA framework balances the core elements to ensure clear rules, timely feedback, and effective emotional guidance. Mechanics are the primary concern for designers, while aesthetics are the first interaction for players. As shown in Figure 2, different arrow colors represent mechanisms that drive user dynamics, complemented by distinct aesthetic elements. The four mechanisms are duck diving background, breathing rhythm control, breath depth and duration, and multidimensional data feedback. These correspond to three dynamics: assuming the yellow duck role, watching and breathing, and controlling the breathing rhythm. Through these dynamics, users experience various aesthetic elements, including fantasy, narrative, sensation, challenge, and emotion.

##### Mechanics

Breathing data guide the duck’s movement—deeper inhalation increases dive depth, breath-holding keeps the duck submerged, and exhalation brings it back to the surface. Positive reinforcement encourages correct breathing, such as rewarding sustained breath-holding and successful dives.

##### Dynamics

Real-time sensor feedback synchronizes the duck’s movements with the child’s breathing rhythm, reinforcing correct behaviors.

##### Aesthetics

Cartoon visuals and lively music enhance interactivity and achievement, with sound effects (quacking and water flow) synchronized to breathing rhythms. The underwater environment, including corals, seaweed, and fish, increases immersion and reinforces breathing control.

### Implementation of the Visual Interaction Layer

**Figure 3.**
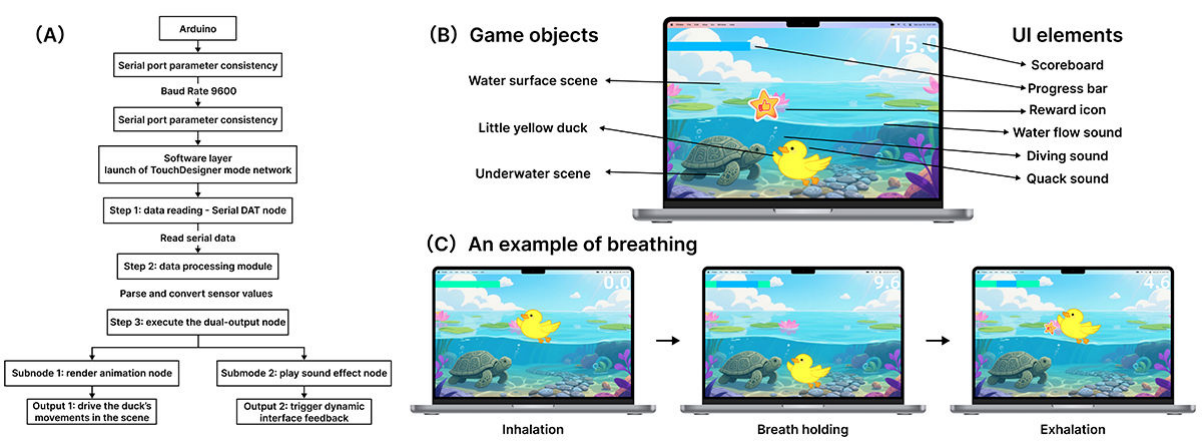
Software implementation. (A) Implementation flow of the BreatheBuddy system's visual interaction layer; (B) using the breath-holding scenario as an example, labeling game objects and user interface elements; and (C) visual interaction screens corresponding to the 3 stages of breathing—inhalation, breath holding, and exhalation. UI: user interface.

The visual interaction layer was constructed using the TouchDesigner platform. TouchDesigner is a real-time, node-based interactive visualization software that enables the creation of both 2D and 3D animations and audio. Its features of “real-time processing” and “visual programming” make it particularly well-suited for developing dynamic game feedback interfaces [49]. In this study, a node network was established in TouchDesigner, consisting of the Serial DAT node for reading serial data, data processing modules, and a rendering system for animations and sound effects. TouchDesigner natively supports Arduino (developed by Haoyu Zhang) data, allowing direct serial data acquisition through the Serial DAT node and enabling synchronization between hardware signals and visual animations [50]. The serial port baud rate was configured to match the Arduino program (9600 baud), and the table format was set to “one row per line.” Under this configuration, each line of sensor values transmitted from the Arduino was received and parsed in real time by TouchDesigner to drive the duck’s movements and interface feedback, as illustrated in Figure 3A.

In terms of visual presentation, the system uses interface elements such as a progress bar and a score panel to display children’s breathing status and training progress in real time (Figure 3B). This provides intuitive feedback on training outcomes and enhances children’s sense of accomplishment. When a child performs the breathing maneuver correctly, the system triggers a sequence of immediate positive feedback, including the duck’s movement, a “successful dive” animation, and corresponding sound effects. This mechanism reinforces correct behaviors and helps sustain the training rhythm.

From the aesthetics dimension of the MDA framework, these audiovisual feedback cues cultivate an enjoyable interactive experience, thereby strengthening children’s affective engagement and motivation to participate.

During software prototyping, multiple rounds of internal testing were conducted to verify animation smoothness and interaction accuracy under real-time breathing-data control. The system can dynamically tailor feedback content and visual effects according to children’s breathing behaviors (Figure 3C), ensuring timely and clear feedback. In doing so, it supports training adherence while increasing the likelihood of sustained participation in breathing training.

### Implementation of the Physical Interaction Layer

The hardware component primarily consists of an Arduino Uno microcontroller board and an airflow sensor. The Arduino Uno is based on the ATmega328P chip, offering 14 digital input and output pins (including 6 supporting pulse-width modulation output) and 6 analog inputs. It is also equipped with a USB interface and a power jack, facilitating convenient connection to computers and external power supplies [51]. The airflow sensor design is inspired by the water flow sensor, ingeniously adapting its working principle to measure airflow. Detailed parameters are provided in Table 1. Conventional airflow detection devices are typically expensive and possess specialized structural designs, limiting their wide application. By leveraging the principles of water flow sensors, airflow can be effectively detected, thereby providing technical support for interactions involving airflow or exhalation.

**Table 1.** Airflow sensor parameters.

Parameters	Specifications
Module dimensions	Length 3 cm x width 2.1 cm
PCB^a^ thickness	1.6 mm
Total module thickness	6 mm
Module weight	55 g (including breath sensor)
Supply voltage	DC^b^ 3.3V/5V
Output signal	Serial port data output, raw pulse signal output, and TTL^c^ level output

a PCB: printed circuit board.

b DC: direct current.

c TTL: transistor-transistor logic.

The sensor consists of a plastic valve body, a flow rotor assembly, and a Hall sensor. When airflow passes through the rotor, the rotational speed is proportional to the flow rate. The Hall sensor generates pulse signals, which are transmitted to the controller for computation, and the processed data are ultimately output via a serial port. In this system, the sensor is seamlessly integrated into the inhaler. Whenever the child inhales or exhales, the airflow passes through the airway, and the sensor continuously monitors the data in real time. The Arduino controller processes this data to measure inhalation speed, breath-holding duration, and exhalation rhythm.

#### Appearance Design

**Figure 4.**
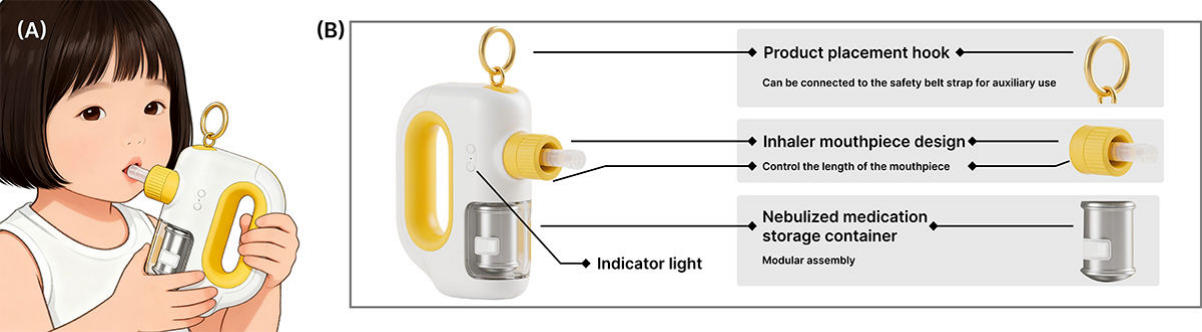
Inhaler design. (A) Rendering scene and (B) side view of the inhaler, illustrating the structure and function of its components.

The appearance design emphasizes child-friendliness and the minimization of a medical atmosphere. Both the inhaler shell and the sensor module adopt rounded shapes to ensure safety during use. The overall color scheme features bright yellow, complemented with cartoon duck elements, making the device resemble a toy rather than a medical instrument, thereby alleviating children’s anxiety during use [31]. Furthermore, the sensor and inhaler body adopt a modular, detachable design, facilitating cleaning, maintenance, and replacement. All edges are rounded to avoid sharp corners, complying with child safety standards, as illustrated in Figure 4A and 4B. The main body of the inhaler measures approximately 12.6 cm (length) × 4.3 cm (width) × 15.2 cm (height).

#### Functional Implementation

**Figure 5.**
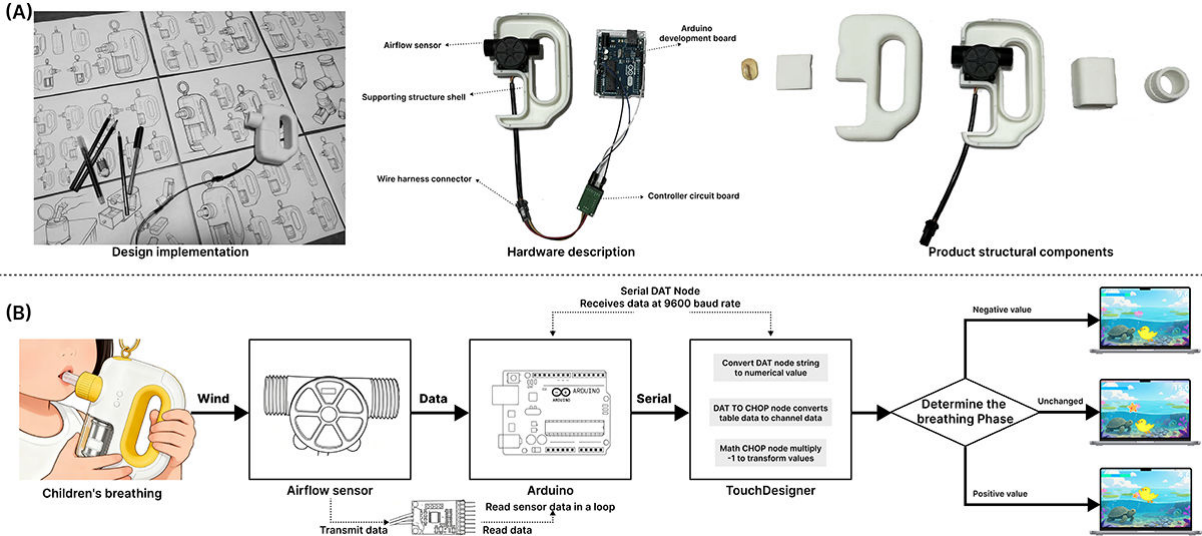
Functional configuration and implementation. (A) Inhaler device prototype: 3D printing, hardware specifications, and structural components, and (B) system functional data flow path.

The functional implementation encompasses both the deployment of the inhaler prototype and the flow of system data, as illustrated in Figure 5A and 5B. On the Arduino side, a program for serial communication and signal processing was developed to collect sensor data and transmit it to the software system. The program initializes the serial port using Serial.begin(9600) and then enters a loop to continuously read values from the airflow sensor. Within each loop iteration, the analog signal from the airflow sensor is read, and the sampled data, along with status information, is sent line by line via the USB serial interface using Serial.print(). In TouchDesigner, a Serial DAT node is configured at a baud rate of 9600 with a “one value per line” table format to receive this data. Through continuous sensor reading, the software layer captures children’s respiratory information.

In this system, airflow sensor output data are transmitted to TouchDesigner in string format by default. To facilitate subsequent mathematical operations and interaction design, the data are converted into numerical values.

To map inhalation values to negative numbers while maintaining exhalation as positive, mathematical operations are applied using a Channel Operator (CHOP). Since DAT stores tabular data, it is first transformed into CHOP format by creating a DAT to CHOP node (datTo1) from convert1 (Create → CHOP → DAT To). In the parameter panel of datTo1, the appropriate column is selected to convert the tabular data into channel data. Subsequently, a Math CHOP node (math1) is created (Create → CHOP → Math). In math1*,* the Operation is set to multiply with the value parameter as “−1,” thereby inverting the original positive values to negative during inhalation. This transformation ensures that inhalation is represented as negative, exhalation as positive, and breath-holding remains unchanged, which in turn drives the dynamic behavior of the virtual duck.

### Research Hypothesis

To address RQ2 (Does the accuracy of children’s inhalation, breath-holding, and exhalation rhythm improve, thereby enhancing therapeutic outcomes?) and RQ3 (Does children’s treatment adherence improve?), this study was designed and implemented a quantitative controlled experiment, referencing the CONSORT (Consolidated Standards of Reporting Trials) reporting guidelines to ensure transparency and clarity in reporting (Checklists1  2 [52]). The experiment compares the differences in respiratory behaviors, psychological experiences, and adherence between the BreatheBuddy system and the inhaler-only control group, to test the following research hypotheses:

- Hypothesis 1: compared with the control condition, children using BreatheBuddy will show significant improvements in inhalation rhythm accuracy, breath-hold duration, and exhalation control, indicating higher effectiveness-related indices of inhalation therapy.
- Hypothesis 2: compared with the control condition, BreatheBuddy will provide superior interactivity and enjoyment, thereby significantly increasing children’s engagement and treatment adherence.
- Hypothesis 3: compared with the control condition, BreatheBuddy will better facilitate immersion and flow, reduce treatment-related anxiety, and improve attentional focus, thus alleviating discomfort during use.
- Hypothesis 4: compared with the control condition, BreatheBuddy will receive higher scores on user evaluation indicators, including usability, playability, and overall satisfaction.

### Eligibility Criteria

Inclusion criteria were children aged 6-8 years with prior experience using inhaler-based therapy. Children in this age range typically have sufficient cognitive and expressive abilities to complete the experimental tasks [53], and prior inhaler experience helps minimize potential confounding effects due to unfamiliarity with device operation. Exclusion criteria covered two groups: (1) children with severe language or cognitive impairments who could not reliably follow the procedure or provide feedback; and (2) children who, within the past month, had respiratory infections, acute asthma exacerbations, or were unable to complete the tasks independently, as these factors could compromise the accuracy of respiratory-behavior measurements and overall data quality.

### Patient and Public Involvement

A total of 20 children were recruited (10 boys and 10 girls), with a mean age of 6.9 (SD 0.79) years. All participants had prior experience with inhaler-based treatment. Sample size determination was guided by effect estimates from a pilot study. Given resource constraints and the practical challenges of recruiting pediatric participants, we adopted a small-sample controlled experimental design focused on children. Future studies may increase the sample size and conduct power analyses to improve statistical precision.

### Sampling Procedures

Participants were recruited through local educational institutions and community organizations. Study information was disseminated via internal announcements. Parents or legal guardians voluntarily registered their children for participation after being fully informed about the study’s purpose and procedures.

### Trial Design

This study used a one-factor repeated-measures experimental design, with the independent variable being the type of intervention, which included 2 conditions assigned with a randomization ratio of 1:1 (ie, each participant received both intervention conditions, ensuring equal exposure to both conditions). This design aimed to minimize the confounding influence of individual differences on the experimental results, thereby enhancing statistical sensitivity and power.

#### Control Condition

Participants used an inhaler that matched the conventional clinical inhaler in hardware structure and operating logic for simulated training. No gamified or interactive feedback was provided; researchers offered operational guidance only when necessary. To improve children’s acceptance, the inhaler’s appearance was slightly “fun-optimized,” without altering its core structure or operation.

#### Experimental Condition

Participants used the same inhaler hardware as in the control condition, retaining all conventional structure and operating steps, but with an added gamified feedback mechanism (the BreatheBuddy training system). Children advanced the game by performing standardized inhalation actions, and the system provided real-time visual and interactive feedback to enhance engagement and adherence. All participants experienced both conditions in a randomized order to reduce the influence of individual differences, increase sensitivity to intervention effects, and improve statistical efficiency. No medication was administered; participants were informed that the tasks involved simulated training for inhaler use. Each intervention lasted 3 minutes. After each intervention, researchers assisted participants with questionnaires and conducted a brief interview.

### Randomization

This study uses a sequential experimental design, where the order of intervention for each participant is determined by their sequence of arrival at the laboratory. Each participant completed all interventions for both the control group and experimental group in the assigned order. To avoid selection bias, the first 10 participants first underwent the experimental group intervention, followed by the control group intervention; whereas the latter 10 participants first underwent the control group intervention, followed by the experimental group intervention.

### Blinding

Due to the significant differences in the interactive nature of the 2 intervention conditions (the experimental group includes gamified feedback, while the control group does not), blinding could not be applied to the participants or the experimental staff. However, blinding was implemented during the data collection and analysis phase. Researchers responsible for data entry and statistical analysis were unaware of the participants’ intervention sequence and processed the data using anonymized codes to minimize measurement and analysis biases.

### Instrumentation

#### Inhaler Device

**Figure 6.**
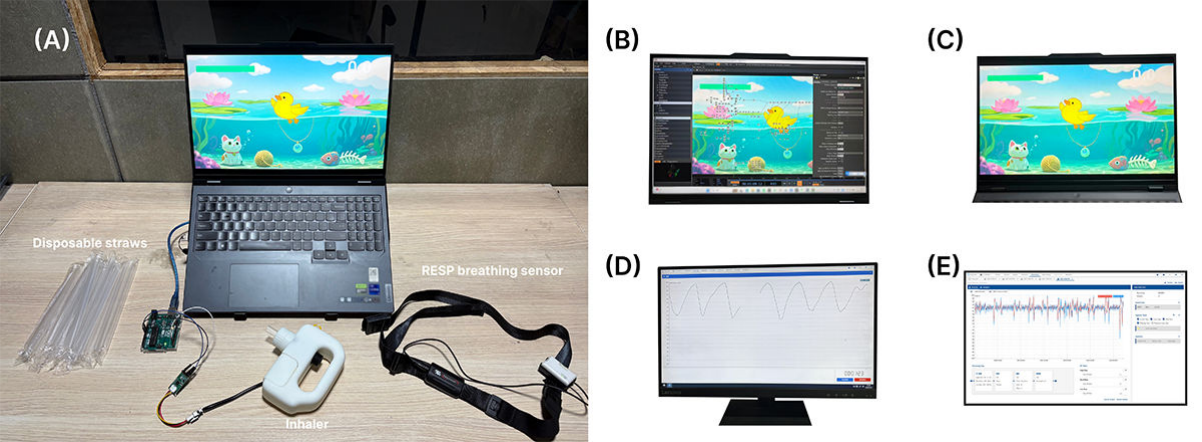
Experimental setup and equipment. (A) Overall experimental materials: replaceable straws, BreatheBuddy system-specific inhaler equipped with airflow sensors, respiration (RESP) sensor for capturing respiratory signals; (B) interactive system configuration; (C) laptop computer loaded with experimental game software for presenting game content and facilitating interaction; (D) respiratory data acquisition device; and (E) RESP respiratory analysis module.

The BreatheBuddy inhaler device was used. It integrates an airflow sensor to record respiratory data and transmits the data via Arduino to an interactive visual module. Disposable, eco-friendly straws were used as mouthpieces to prevent cross-contamination (Figure 6A).

#### Data Acquisition Devices

A Lenovo Y9000P (2023) laptop displayed the experimental game content (Figure 6B and 6C). An iPhone was used to record semistructured interviews. A respiration sensor was used to collect real-time changes in respiratoryrhythm (Figure 6D and 6E).

#### Questionnaire Tools

Electronic questionnaires were created on Wenjuanxing and included the following standardized scales:

1. Player Experience of Need Satisfaction (PENS): the relatedness and belonging subscale was removed; autonomy, competence, immersion, and intuitive controls were retained (20 items), rated on a 7-point Likert scale.
2. Game User Experience Satisfaction Scale (GUESS): 7 dimensions (eg, creative freedom and enjoyment; 26 items), rated on a 7-point Likert scale.
3. System Usability Scale (SUS): 6 selected items, rated on a 5-point Likert scale.

### Data Collection

The procedure consisted of four stages, lasting approximately 46‐56 minutes in total:

1. Preparation (10‐15 minutes): researchers explained the study purpose, procedures, and risks to participants and guardians, obtained written consent, helped participants adapt to the setting, prepared disposable straws, and installed them on the inhaler.
2. Task execution (16 minutes): participants completed training tasks under both conditions; each task lasted 3 minutes. A 10-minute recovery period was provided between the 2 interventions. Condition order was randomized to control for order effects.
3. Questionnaire and interview (15‐20 minutes): after each intervention, researchers assisted participants in completing the electronic questionnaires and conducted semistructured interviews. Interviews focused on subjective experience and perceived mastery of breathing accuracy, while also collecting caregiver observations and expert feedback regarding clinical applicability.
4. Completion (5 minutes): researchers thanked participants and guardians and provided a small yellow duck–themed gift.

### Quality of Measurements

Standardized data collection was implemented during questionnaire completion, during which researchers provided age-appropriate explanations and support to address limitations in children’s literacy and expression, ensuring valid feedback data [54]. Scale quality assurance was ensured as PENS, GUESS, and SUS are well-established instruments in psychology and user-experience research with good reliability and validity, enabling robust assessment of children’s intrinsic motivation, game experience, and system usability. Device calibration was conducted before the experiment, with all equipment rigorously calibrated and tested to ensure measurement accuracy and stable operation.

### Data Diagnostics

Respiratory behavior data were denoised using Gaussian smoothing and downsampling to improve signal stability. Peaks and troughs were detected to determine the start and end of each breathing cycle. Each cycle was segmented into inhalation, exhalation, and breath-hold phases, and phase durations were computed. Breathing rate was calculated from the time differences between adjacent peaks, and breathing depth was estimated from peak amplitude differences. This study did not perform missing-data imputation or outlier removal.

### Analytic Strategy

Quantitative analysis was conducted on respiratory behavior, and scale data were summarized using descriptive statistics. Paired-samples *t* tests were used to compare the 2 conditions; if normality assumptions were violated, the Mann-Whitney *U* test was used as a nonparametric alternative. Qualitative analysis was conducted as semistructured interviews were transcribed and analyzed using thematic analysis to extract key themes related to breathing accuracy, flow experience, and system usability, which were used to complement and help interpret the quantitative findings.

### Qualitative Methods

#### Research Design Overview

The qualitative component of this study was embedded within a mixed method randomized crossover experimental design. Semistructured interviews were conducted to gain an in-depth understanding of children’s subjective experiences under different intervention conditions, including their perceived mastery of breathing rhythm, emotional responses, sense of immersion, and evaluations of system usability.

Within the overall research framework, the qualitative inquiry served an explanatory function, complementing and interpreting the quantitative findings to enhance the comprehensiveness and interpretive depth of the study’s conclusions.

#### Study Participants or Data Sources

Qualitative data were collected from all 20 child participants who completed the experimental tasks. Each participant took part in a semistructured interview after completing both intervention conditions. In addition, caregivers’ observational feedback was documented during the interviews, and expert opinions regarding the system’s clinical applicability were collected to supplement the children’s self-reported data. All interviews were audio-recorded and transcribed verbatim after the experiment for subsequent analysis.

#### Participant Recruitment

The qualitative sample was identical to that of the quantitative experiment. All children who completed the full experimental procedure were invited to participate in the interviews, with no additional inclusion or exclusion criteria. Written informed consent was obtained from parents or legal guardians for the qualitative data collection procedures, and children provided verbal assent after receiving age-appropriate explanations from the researchers.

#### Data Collection

Semistructured interviews were conducted immediately after each intervention session, with each interview lasting approximately 10‐15 minutes. Interviews were carried out in a quiet laboratory setting. The interview protocol focused on the following topics: perceived ease of system use, perceived ease of mastering breathing rhythm, levels of enjoyment or relaxation, willingness to continue using the system, and experiential differences between the 2 intervention conditions.

Interviews were conducted by researchers experienced in communicating with children. When necessary, a guardian was present to enhance the child’s sense of comfort and security during expression. Data collection continued until thematic saturation was reached, defined as the point at which no new themes or concepts emerged in consecutive interviews.

#### Analysis

Following transcription, interview data were analyzed using thematic analysis. The analytic process involved familiarization with the data, initial open coding, clustering similar codes into candidate themes, reviewing and refining themes, defining and naming themes, and integrating themes into an interpretive narrative.

To enhance analytical rigor, 2 researchers independently coded the data and resolved discrepancies through discussion until consensus was achieved. The resulting codes and themes were used to complement the quantitative findings and to strengthen the explanatory depth of the mixed methods design.

### Ethical Considerations

This study was reviewed and approved by the Ethics Committee of Hubei University of Technology (approval no HBUT20250043) and was conducted in accordance with established ethical guidelines for research involving human participants. Before the commencement of the study, written informed consent was obtained from the parents or legal guardians of all participating children. With parents present, researchers explained the experimental procedures to the children in an age-appropriate manner and obtained their verbal assent. Participation was entirely voluntary, and children were free to withdraw from the study at any time without any consequences. All participant data were anonymized and deidentified to prevent any linkage to personally identifiable information. Data were accessible only to the research team and were handled with strict confidentiality. All images included in this paper and its additional materials were carefully reviewed to ensure that no individual participant could be identified and that no personally identifiable information was disclosed. No monetary compensation was provided. Upon completion of the experiment, each child received a themed “Little Yellow Duck” card as a commemorative token. The experiment was conducted in the User Experience and Interaction Design Laboratory at Hubei University of Technology. The laboratory is equipped with a one-way mirror, allowing observation and supervision of procedural compliance and participant status to ensure the safety and integrity of the experimental process.

## Results

### Overview

To ensure comparability between the experimental and control groups on the same scale, we standardized the scores of the PENS, GUESS, and SUS to a range of 1 to 100 (standardized scores [SSs]). The Shapiro-Wilk test was performed to assess the normality of the data [55]. For data that did not follow a normal distribution, the Mann-Whitney *U* test was used to evaluate the differences between the 2 groups regarding breathing behavior and user experience [56]. For data that followed a normal distribution, paired *t* tests were conducted to compare the differences between the 2 groups.

This study involved 20 participants, a sample size chosen based on the design and effect size of previous similar studies. Although the sample size is relatively small, significant differences were observed between the experimental and control groups, which were consistently statistically significant across all indicators (*P*<.05). Furthermore, the 95% CIs for all results did not include 0, further confirming the reliability of the differences between the groups. These findings suggest that, despite the limited sample size, the results are statistically significant, supporting the effectiveness of the proposed system in improving inhaler accuracy and adherence.

**Figure 7.**
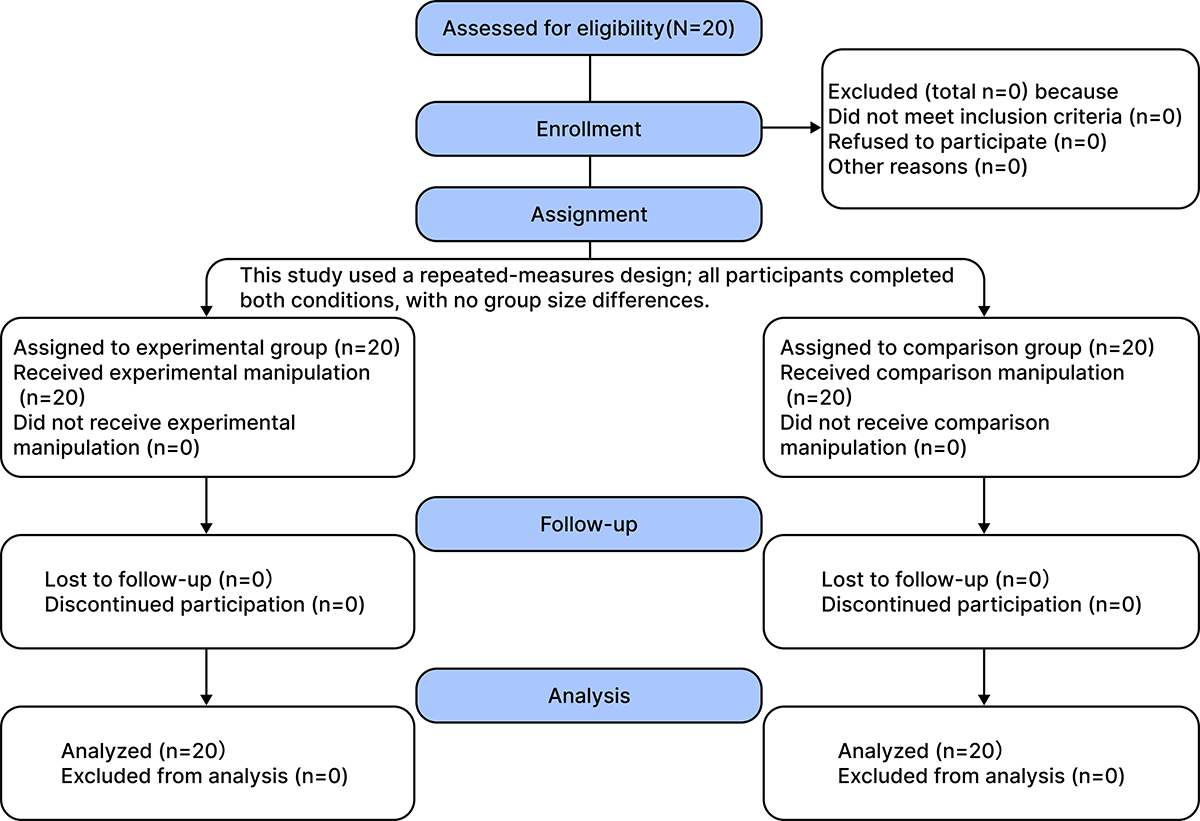
Participant flowchart for a repeated-measures controlled experiment comparing BreatheBuddy and standard inhaler training in children.

The progression of participants across each stage of the study is illustrated in the participant flowchart (Figure 7). All 20 participants completed both intervention conditions.

### Accuracy of Respiratory Behavior

To facilitate comparison, we compared the longest breath-holding durations between the experimental group and the control group. The results showed that the data followed a normal distribution, with the experimental group’s average longest breath-holding duration being 12.20 (SD 2.11 seconds; 95% CI 11.263-13.131 seconds), which was longer than the control group’s 7.92 (SD 2.27 seconds; 95% CI 6.909-8.921 seconds), with a significant paired *t* test (*P*<.001). The control group, which used a traditional inhaler, demonstrated significantly shorter breath-holding durations. These findings suggest that after using the “BreatheBuddy” system, the experimental group was better able to maintain breath-holding time, showing both longer durations and more stable breathing rhythms.

Further time-domain analysis revealed that the data followed a normal distribution, with the experimental group’s respiration data showing smaller variance and SD, which was statistically significant (paired *t* test; *P*<.001). This indicates that the experimental group exhibited more stable breathing rhythms. The smaller standard deviation and variance further support the advantages of the experimental group in terms of breath-holding duration and rhythm stability, demonstrating superior performance in breathing training. Additionally, the experimental group’s average breathing frequency was 9.97 (SD 0.57 rpm; 95% CI 9.719-10.229 rpm), significantly lower than the control group’s 10.47 (SD 0.82 rpm; 95% CI 10.110-10.837 rpm; paired *t* test; *P*=.03). The experimental group’s minimum breathing frequency was 4.63 (SD 0.78 rpm; 95% CI 4.284-4.973 rpm), significantly lower than the control group’s 6.02 (SD 1.15 rpm; 95% CI 5.508-6.525 rpm; paired *t* test; *P*<.001). These results indicate that the experimental group exhibited more stable and relaxed breathing patterns, further confirming its superiority in breath control and breath-holding stability.

### User Motivation and Need Satisfaction

**Figure 8.**
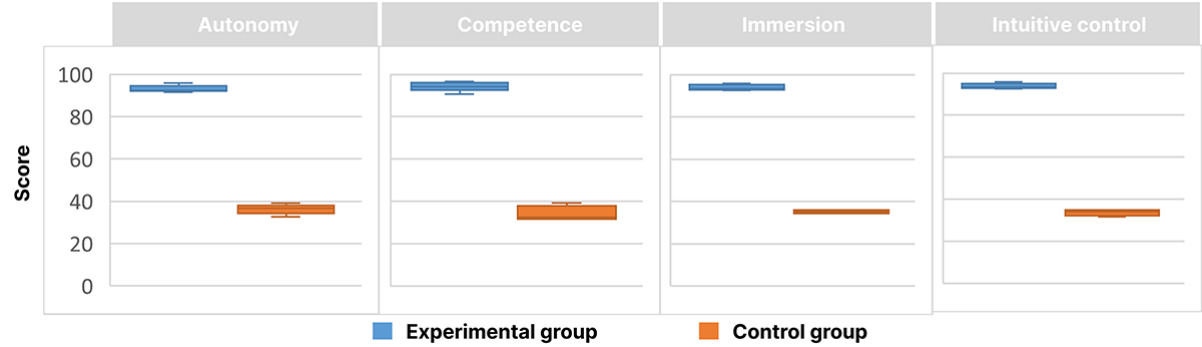
Evaluation and comparison of Player Experience of Need Satisfaction (PENS) for 2 groups across 4 dimensions.

The PENS questionnaire data followed a normal distribution, and paired *t* tests were used to compare the scores between the 2 groups, revealing a significant difference (*P*<.001). After standardization, the experimental group (SS 93.833, 95% CI 92.819‐94.847) scored significantly higher than the control group (SS 34.792, 95% CI 31.805‐37.778). The results indicate that the experimental group participants scored significantly higher on the PENS questionnaire compared to the control group and outperformed the control group in the dimensions of autonomy, competence, immersion, and intuitive control, as shown in Figure 8.

### User Experience Satisfaction

**Figure 9.**
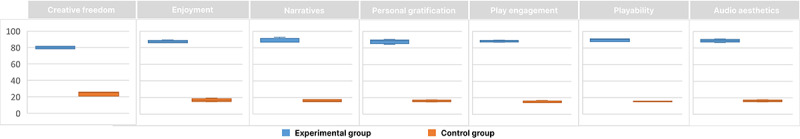
Evaluation and comparison of Game User Experience Satisfaction Scale (GUESS) across 7 dimensions for 2 groups.

As the overall scores of the GUESS questionnaire did not follow a normal distribution, we used the Mann-Whitney *U* test to compare the median score differences between the experimental group (median 87.917, IQR 86.54-88.46) and the control group (median 15.833, IQR 15.38-19.23). The test results revealed a statistically significant difference between the 2 groups (*P*<.001). The results indicated that the experimental group scored significantly higher than the control group on the GUESS questionnaire and outperformed the control group across all 7 dimensions (refer to Figure 9).

### System Satisfaction

The SUS questionnaire results followed a normal distribution. An independent samples *t* test revealed a significant difference between the 2 groups (*P*<.001). The experimental group (SS 88.958, 95% CI 86.394-91.522) scored significantly higher than the control group (SS 20.833, 95% CI 18.844-22.823). These results indicate that participants in the experimental group reported substantially higher system satisfaction compared to those in the control group.

### Interview Findings

During the analysis of interview data, the research team iteratively developed and refined a coding framework based on feedback from children, caregivers, and experts. Five core themes were ultimately identified: (1) intrinsic motivation and behavioral change, (2) flow experience and engagement, (3) game mechanics and playability, (4) feedback mechanisms and breathing accuracy, and (5) caregiver and expert perspectives. The detailed findings are presented in the following 5 subheadings.

### Intrinsic Motivation and Behavioral Change

Most children reported that BreatheBuddy helped them understand the training goals, which became increasingly clear over time. Although the quantitative data did not reveal significant differences, this qualitative feedback remains noteworthy. Children found the system’s instructions and tasks easy to follow, which facilitated mastery of the inhalation, breath-holding, and exhalation sequence. For example, one child stated, *“*I know what to do in each training session because the screen shows me.*”* Another commented: *“*I like seeing the progress bar—I want to hold my breath longer so the little duck can dive deeper.” Some children, however, felt that the tasks were insufficiently challenging. As one noted: *“*Some tasks are too easy and I finish them quickly—it doesn’t feel difficult enough.” This suggests that adjusting task difficulty appropriately may enhance children’s motivation.

### Flow Experience and Engagement

The majority of children reported that they could maintain concentration during training, particularly when interacting with the “duck diving” animation and receiving real-time feedback, which enhanced their sense of immersion and control. One child remarked, *“*I like watching the duck dive; it feels like I’m diving too.*”* Another explained: *“*When the system tells me I’m doing it right, I focus more.*”* However, a few children noted that task repetition sometimes reduced their attention. One participant shared: *“*Doing it too many times gets boring—I want to level up.*”* These findings suggest that balancing enjoyment with novelty is important to prevent monotony-induced disengagement.

### Game Mechanics and Playability

The gamified design of the system, especially the “duck diving” interaction, was highly appealing to children. Most reported that the animations and auditory feedback enhanced both the enjoyment and attractiveness of the training. One child noted: *“*Every time I see the duck dive, I know I did it right.*”* Another added: *“*I like watching the duck dive—it keeps me from getting distracted.*”* The system’s use of animation and diving tasks helped children sustain attention for extended periods, transforming breathing training from a monotonous activity into a playful and enjoyable experience.

### Feedback Mechanisms and Breathing Accuracy

Most children believed the system’s feedback mechanisms supported their gradual mastery of breathing accuracy, with the visual and auditory feedback in the “duck diving” task proving particularly effective. One child explained: *“*When I inhale, the duck dives under the water, and there’s also a sound reward.*”* Another said: *“*By watching what the duck does, I can adjust my breathing.*”* This type of real-time feedback helped children correct errors and improve control over inhalation, breath-holding, and exhalation. Nevertheless, the depth of personalization in feedback requires further enhancement to better suit children at different skill levels. As one child suggested: *“*I hope the feedback could be more varied, based on my performance.*”*

### Caregiver and Expert Perspectives

Parents generally observed that their children showed high levels of interest and positive emotions while using the system and were able to complete the training as required. One parent shared: *“*My child was very engaged when using it—not anxious at all, and actually enjoyed the process.” Another added: “I noticed he could follow the instructions more attentively, and his breathing became steadier than before.” Experts highlighted the system’s clinical potential for attracting children’s attention, enhancing engagement, and improving breathing accuracy. One expert commented: *“*The gamified design is highly appealing to children and increases their willingness to cooperate with training.” However, experts also recommended improvements, such as introducing a progressive difficulty system to accommodate varying ages and skill levels, thereby better supporting skill development.

## Discussion

### Principal Findings

This study focuses on the core research goal outlined in the introduction: to develop and assess an asthma inhaler training system, BreatheBuddy, which integrates gamified feedback to improve inhalation techniques and treatment adherence in children aged 6-8 years with asthma. The study also addresses 3 RQs (ie, RQ1-RQ3). Using a single-factor repeated-measures controlled experiment, the research systematically evaluates the practical effectiveness of this system in pediatric inhalation therapy. In line with the proposed research hypotheses 1-4, the main findings of this study are as follows: the BreatheBuddy system significantly improves inhalation rhythm accuracy, extends breath-holding duration, and stabilizes breathing patterns. It also enhances treatment adherence, engagement, and satisfaction while reducing anxiety related to the treatment process. Moreover, the system outperforms traditional inhaler training methods in terms of usability and playability. By embedding breathing behavior into the game interaction mechanism, BreatheBuddy achieves dynamic mapping of the breathing rhythm to real-time feedback, seamlessly integrating skill training, motivation enhancement, and emotional regulation. This addresses the key issues of RQ2 and RQ3 regarding improvements in inhalation accuracy and adherence. It also provides an empirically validated design paradigm for answering RQ1’s question: “How can an interactive inhaler training tool with a gamification system be designed for children?”

### Results Interpretation and Comparison With Literature

This study’s primary findings focus on 3 key aspects: the standardization of respiratory behavior, the stimulation of intrinsic motivation, and the optimization of the gaming experience. These results not only align with existing research but also represent innovative breakthroughs in specific research contexts, further expanding the field of gamified health interventions and pediatric asthma treatment.

Regarding respiratory behavior accuracy and treatment effectiveness, this study demonstrates that BreatheBuddy effectively helps children develop a standardized and stable “inhalation-holding-exhalation” rhythm, particularly excelling in extending breath-holding duration and enhancing the stability of breathing patterns. Numerous studies have confirmed that breath-holding duration, inhalation depth, and respiratory rhythm stability are crucial factors influencing the efficiency of pulmonary drug deposition, directly determining the quality of treatment outcomes [57-59]. These findings are highly consistent with the results of this study, further confirming the potential of improving children’s inhalation techniques through behavioral guidance. Unlike previous research that primarily relied on verbal guidance from health care professionals, passive demonstrations, or simple visual prompts for training [46-48], BreatheBuddy innovatively integrates respiratory behavior with visual and interactive gaming feedback. This allows children to immediately assess whether their actions meet treatment standards during the process, enabling them to correct their actions without additional cognitive burden. This approach is in line with Zainal et al’s [60] assertion that “immediate feedback is the core driver of health behavior change.” However, this study goes beyond existing research by not only providing prompts for a single respiratory action but also integrating feedback mechanisms throughout the entire respiratory cycle, thus guiding inhalation, breath-holding, and exhalation. This comprehensive feedback system is a key factor in the significant success of this study in standardizing respiratory behavior. Moreover, traditional inhaler training often neglects the development of children’s awareness of body rhythm and control over their movements, making it difficult to maintain training effectiveness [24,61]. In contrast, BreatheBuddy, through its design combining a physical inhaler with visual gaming feedback, provides children with clear rhythm cues and behavioral guidelines, significantly improving the consistency and stability of their breathing operations. This suggests that multimodal feedback may be more effective than single-modality interventions for helping children master complex therapeutic actions, offering a promising new approach for pediatric inhaler training.

In terms of intrinsic motivation and treatment adherence, this study found that BreatheBuddy effectively enhances children’s intrinsic motivation and boosts their engagement in treatment. This aligns well with self-determination theory, which emphasizes that “autonomy, competence, and immediate feedback are the core elements of intrinsic motivation” [44,62]. Through features such as real-time animated feedback and progress visualization, the system enables children to clearly understand the link between their efforts and the results, helping them gradually develop a sense of control over their inhalation treatment, thus reducing resistance or negative emotions. Unlike some gamified health interventions that overly rely on external rewards, such as points or badges, to maintain participation [63], this study observed that BreatheBuddy fosters a shift from external rewards to intrinsic motivation. Children began to view “regulating their breathing to advance the game” as an inherently rewarding activity, rather than simply a burdensome treatment task. This finding supports Michaelsen and Esch’s [64] conclusion that “short-term rewards can spark motivation, but long-term treatment adherence depends on cultivating intrinsic motivation.” In contrast to traditional inhaler training, which lacks interactivity and contextual feedback, often leading to resistance and distraction in children [65], BreatheBuddy’s gamified design effectively addresses these challenges, significantly increasing children’s willingness to engage. This result is consistent with previous research on gamified interventions in pediatric health care [66] and contributes new empirical evidence in the specific context of inhaler training, offering fresh insights into improving adherence in pediatric chronic disease treatment.

This study demonstrates that BreatheBuddy outperforms traditional training methods in terms of immersion, playability, and overall user experience. These findings are consistent with the core principles of the MDA game design framework [45], which emphasizes the integration of entertainment and therapeutic effectiveness through optimization of the 3 key components: mechanics, dynamics, and aesthetics. Previous research has shown that immersive experiences can help users maintain focus during tasks, reduce discomfort sensitivity, and encourage sustained participation [67]. The results of this study further support this notion: BreatheBuddy, with its “Little Yellow Duck Diving” narrative, synchronized audiovisual feedback, and adaptive task difficulty, transforms the monotonous process of breathing exercises into an engaging game-like scenario with clear goals. This approach helps children maintain focus while reducing treatment-related anxiety. These results align with the findings of Lockwood et al [68], who concluded that “gamified interfaces can enhance emotional experiences and reduce anxiety in pediatric medical interventions.” Furthermore, this study highlights that in medical training, a positive gaming experience not only provides emotional benefits but also helps standardize breathing behaviors by sustaining attention. However, some children reported in interviews that repeated tasks over time could diminish the sense of immersion, which reflects the well-documented issue of “diminishing novelty” in gamified interventions [69]. This feedback offers valuable insights for future system optimization.

### Limitations

Although this study has validated the effectiveness of the BreatheBuddy system in enhancing children’s inhalation skills, treatment adherence, and overall user experience, several limitations exist in the research design and implementation process that require further refinement in future studies. First, the sample size of this study is limited to 20 children, with a relatively narrow age range (6‐8 years), all participants being recruited from local community health centers and kindergartens. This limited sample size reduces the representativeness of the study and may impact the generalizability of the results, making it difficult to extrapolate the findings to younger children (aged <6 years) or older children (aged >8 years). Furthermore, the study does not fully capture the experiences of children from different regions or socioeconomic backgrounds. Second, the study used a short-term intervention design without long-term follow-up, preventing the assessment of the system’s sustained impact on children’s inhalation techniques, treatment adherence, and overall effectiveness in daily use over time. The study also does not evaluate the system’s impact on asthma clinical outcomes, such as the frequency of acute exacerbations or lung function improvements. Long-term adherence and clinical outcomes remain key objectives in pediatric asthma management [9]. Additionally, while the gamification mechanism effectively motivates children’s engagement, it may inadvertently pose risks: certain children may become overly focused on achieving “excellent performance” in the game, such as holding their breath for excessive durations, thus compromising the comfort and safety of the breathing process. This is especially concerning for children with compromised lung function, as it could exacerbate respiratory strain. This potential risk was not specifically monitored in the current study and warrants further investigation in future research. Finally, while the gamification design was tailored to children’s psychological traits, it did not sufficiently account for individual differences, such as variations in personality or cognitive abilities. This lack of personalized adaptation may limit the system’s effectiveness across diverse child populations.

### Conclusion

This study empirically evaluated the effectiveness of the BreatheBuddy system in pediatric asthma inhalation therapy through a one-factor repeated-measures controlled experiment, successfully addressing the core RQs and achieving the research objectives. The primary innovation lies in the seamless integration of breathing rhythm with dynamic game interaction, facilitating the synergy of skill development, motivation enhancement, and emotional relief. This approach effectively addresses challenges related to improper inhalation techniques and poor adherence in children, offering an innovative behavior-guided paradigm for pediatric asthma inhalation training.

Academically, this study expands the scope of gamified health interventions in pediatric chronic disease management, providing new empirical evidence on the role of interactive design in promoting treatment adherence among children. Practically, the system is user-friendly, child-centered, and adaptable to both home and clinical environments, thereby improving long-term asthma treatment outcomes and self-management capabilities. The research findings also offer valuable insights for the treatment of other pediatric chronic conditions. Future efforts to expand the sample size, conduct long-term follow-ups, and optimize the system design will further enhance its clinical application value.

## Supplementary material

10.2196/85673 Multimedia Appendix 1 Guidelines for correct use of different inhaler devices, including device types, usage steps, and instructions.

10.2196/85673 Checklist 1 CONSORT checklist.

10.2196/85673 Checklist 2 CONSORT-eHEALTH (V 1.6.1) checklist.

## Abbreviations

CHOP: Channel Operator
CONSORT: Consolidated Standards of Reporting Trials
GUESS: Game User Experience Satisfaction Scale
MDA: Mechanics, Dynamics, and Aesthetics
PENS: Player Experience of Need Satisfaction
RQ: research question
SS: standardized score
SUS: System Usability Scale
