# Supplementary material for: Gamified Feedback-Based Training System for Pediatric Asthma Inhaler Use: Mixed Methods Randomized Crossover Study
Source: JMIR Serious Games. 2026 May 4;14:e85673. doi: 10.2196/85673 (PMC13138708; doi:10.2196/85673)
Supplement: Multimedia Appendix 1 [file games-v14-e85673-s001.docx]

Guidelines for Correct Use of Different Inhaler Devices: Including Device Types, Usage Steps, and Instructions

| **Inhaler Type** | **Steps** | **Operating Instructions** |
| --- | --- | --- |
| **DPI** | 1. Preparation | 1. Hold the casing and push the handle outward, ensuring the slide bar clicks into place and displays the medication dose. |
|  | 2. Exhalation Preparation | 2. Before inhaling, move the device away from your mouth and exhale as completely as possible. |
|  | 3. Inhalation | 3. Inhale steadily and forcefully to ensure the flow rate meets the standard. |
|  | 4. Breath-holding | 4. Hold your breath for 10 seconds after inhaling to allow the medication to settle. |
|  | 5. Slow Exhalation | 5. After holding your breath, exhale slowly. |
|  | 6. Device Closure | 6. Close the device after use, listen for the “click,” and rinse your mouth. |
| **pMDI** | 1. Open the lid and shake well | 1. Open the inhaler cap and shake the medication well. |
|  | 2. Exhalation Preparation | 2. Exhale completely to clear your lungs. |
|  | 3. Inhalation | 3. Place the mouthpiece in your mouth, press down on the device, and inhale slowly to ensure the medication enters your lungs. |
|  | 4. Breath-holding | 4. Hold your breath for 10 seconds after inhaling. |
|  | 5. Exhale slowly and close the protective cap | 5. Exhale slowly after holding your breath, then replace the inhaler cap. |
| **pMDI + Spacer** | 1. Shake the medication well. | 1. Shake the medication well to ensure it is evenly mixed. |
|  | 2. Connect the spacer. | 2. Insert the nozzle into the opening of the spacer. |
|  | 3. Practice taking a deep breath. | 3. Practice taking deep breaths in and out to prepare for controlling your breathing rhythm. |
|  | 4. Inhale the medication. | 4. Place the mouthpiece in your mouth, press the device, and inhale slowly. |
|  | 5. Breath-holding and repeat. | 5. Hold your breath for 10 seconds. Repeat inhaling and exhaling 4-5 times until all medication is inhaled. |
|  | 6. End the procedure. | 6. Exhale through your mouth and rinse your mouth to prevent medication residue. |
| **pMDI + Spacer + Facemask** | 1. Shake medication well | 1. Shake the medication well to ensure it is evenly mixed. |
|  | 2. Connect device | 2. Insert the nozzle into the opening of the spacer. |
|  | 3. Practice deep breathing | 3. Take a deep breath in and a deep breath out to help familiarize yourself with the breathing rhythm. |
|  | 4. Put on mask | 4. Securely fit the mask over your face, press the device, and inhale slowly. |
|  | 5. Hold breath and breathe | 5. Continue holding the mask for 10-15 seconds while taking 6 deep breaths to ensure medication deposition. |
|  | 6. End procedure | 6. After removing the device, exhale slowly through your mouth. Rinse your mouth to prevent medication residue. |
