# Supplementary material for: Gamified Feedback-Based Training System for Pediatric Asthma Inhaler Use: Mixed Methods Randomized Crossover Study
Source: JMIR Serious Games. 2026 May 4;14:e85673. doi: 10.2196/85673 (PMC13138708; doi:10.2196/85673)
Supplement: Checklist 1 [file games-v14-e85673-s002.pdf]

CONSORT Checklist for Reporting Randomized Crossover Trials

Section/topic	Item No	Description	Study-Specific Content
**Title**	1a	Identification as a randomised crossover trial in the title	Although the title does not explicitly identify the study as a randomized crossover trial, the methods section of the main text clearly specifies it as a single-factor repeated-measures crossover trial, which is consistent with the crossover trial design implemented in this study.
**Abstract**	1b	Specify a crossover design and report all information outlined in table 2	A crossover design is clearly specified, and all relevant information outlined in Table 2 (including intervention details, period settings, washout period, and randomization) has been reported in detail in the methods section of the main text.
**Introduction**	2a	Scientific background and	Asthma is a common chronic

Background		explanation of rationale	respiratory disease among children worldwide, and inhalation therapy is the first-line treatment modality. However, children often exhibit incorrect inhaler use and poor treatment compliance, which compromise treatment efficacy. Studies have shown that only 8%-22% of children can use inhalers correctly, and about 74.7% of asthmatic children show non-compliant behaviors during treatment. Although existing interventions such as distraction techniques and inhaler training have certain effects, they have limitations such as high resource investment. Therefore, there is an urgent need to develop interventions to improve inhalation skills and treatment adherence.
Objectives	2b	Specific objectives or hypotheses	The purpose of this study was to develop and evaluate BreatheBuddy, a gamified feedback-

			based training system, for improving the inhalation skills and treatment compliance of children with asthma. Four research hypotheses (H1–H4) were proposed to verify the efficacy of the system: H1 focuses on the improvement of breathing accuracy; H2 on treatment compliance and participation; H3 on anxiety reduction and attention improvement; H4 on system usability and satisfaction.
**Methods:**<br>Trial design	3a	Rationale for a crossover design. Description of the design features including allocation ratio, especially the number and duration of periods, duration of washout period, and consideration of carry over effect	Rationale for the crossover design: This design minimizes the interference of individual differences on experimental results and enhances the sensitivity and statistical power of the findings. Design features: A 1:1 allocation ratio was adopted, with each participant receiving both interventions; the trial consisted of 2 periods (1 period for the experimental

			group and 1 period for the control group), each lasting 3 minutes; a 10-minute washout period (recovery time between the two interventions) was implemented to effectively reduce the residual effect of the prior intervention on the subsequent one and mitigate carry-over effects. The main purpose of setting the washout period is to avoid the impact of the first intervention on the second intervention, ensuring the independence of the two intervention results.
Settings and location	3b	Settings and locations where the data were collected	User Experience and Interaction Design Laboratory, Hubei University of Technology, China. The laboratory is equipped with a one-way mirror to observe and supervise the experimental standardization and participants' status, ensuring the safety and compliance of the experimental process.

Change from protocol	4a	Important changes to methods after trial commencement (such as eligibility criteria), with reasons	No important modifications were made to the study methods after trial initiation.
Participants	4b	Eligibility criteria for participants	Inclusion criteria: Children aged 6–8 years with prior experience in inhaler use. This age group's cognitive level and expressive ability can meet the requirements of experimental tasks, and previous inhaler use experience can eliminate the interference of operational unfamiliarity on experimental results. Exclusion criteria: Children with severe language or cognitive impairment, recent (within 1 month) respiratory tract infection or acute asthma exacerbation, or those unable to complete the experimental tasks independently. These situations may affect the accuracy of breathing behavior measurement and the quality of

			experimental data.
Interventions	5	The interventions with sufficient details to allow replication, including how and when they were actually administered	① Control group: Participants performed simulated training using an inhaler consistent in structure and operation with traditional clinical inhalers, without gamified feedback; operational guidance was provided only when necessary. The inhaler's appearance was moderately optimized for fun to enhance children's acceptance, and each intervention session lasted 3 minutes. ② Experimental group: Participants used the same inhaler hardware as the control group, supplemented with the BreatheBuddy gamified feedback system. They drove the game process through standardized inhalation actions, and the system provided real-time visual and interactive feedback (based on the "little yellow duck diving" narrative framework,

			mapping inhalation, breath-holding, and exhalation to the duck's diving, staying underwater, and floating up). Each intervention session lasted 3 minutes. Both interventions were simulated inhalation training without the use of actual medications. The inhaler used a disposable environmental protection straw as the mouthpiece to avoid cross-contamination.
Outcomes	6a	Completely defined prespecified primary and secondary outcome measures, including how and when they were assessed	① Primary outcome measures: Inhalation rhythm accuracy, breath-holding duration, and exhalation control (assessed via real-time breathing data collection using RESP breathing sensors; Gaussian smoothing and downsampling methods were used for denoising during data preprocessing, and peaks and valleys of breathing data were detected to determine the start and end of each breathing cycle). ② Secondary outcome

			measures: Treatment compliance and participation enthusiasm (assessed using the PENS scale, excluding the "relevance/belonging" dimension, retaining 4 dimensions and 20 items), game experience satisfaction (assessed using the GUESS scale, 7 dimensions and 26 items), system usability (assessed using the SUS scale, 6 items), anxiety levels, and attention concentration (assessed via semi-structured interviews). All indicators were evaluated immediately after each intervention; scales were completed with the assistance of researchers, and interviews were conducted concurrently. The scores of PENS, GUESS, and SUS were standardized to the range of 1 to 100 for comparison.
Changes to outcomes	6b	Any changes to trial outcomes after the	No changes were made to the trial

		trial commenced, with reasons	outcomes after trial initiation.
Sample size	7a	How sample size was determined, accounting for within participant variability	The sample size was determined based on effect estimates from a pilot study, with a total of 20 children recruited (10 boys and 10 girls, mean age 6.9 years, SD = 0.79). Due to the limitations of research resources and the challenges in recruiting child participants, a small-sample controlled design was adopted. Future studies should expand the sample size and improve statistical precision through power analysis.
Interim analyses and stopping guidelines	7b	When applicable, explanation of any interim analyses and stopping guidelines	Not applicable. No interim analyses were conducted, and no stopping guidelines were established for this study.
Randomisation<br>Sequence generation	8a	Method used to generate the random allocation sequence	The random allocation sequence was generated based on the order of participants' arrival at the laboratory. The first 10 participants received the experimental

			intervention first, followed by the control intervention; the subsequent 10 participants received the control intervention first, followed by the experimental intervention, to ensure balanced allocation of the two intervention sequences and eliminate selection bias.
Sequence generation	8b	Type of randomisation; details of any restriction (such as blocking and block size)	Simple randomization was adopted without stratification, and a block size of 10 was used to ensure that 10 participants were assigned to each of the two intervention sequences (experimental group first then control group, control group first then experimental group).
Allocation concealment mechanism	9	Mechanism used to implement the random allocation sequence (such as sequentially numbered containers), describing any steps taken to conceal the sequence until interventions were	The random allocation sequence was implemented via a preset allocation order. The research team pre-determined the intervention order for the first 10 and subsequent 10 participants to

		assigned	ensure that the allocation sequence was not disclosed prior to intervention implementation, thereby avoiding selection bias. The experimental implementers assigned the intervention sequence in accordance with the preset order.
Implementation	10	Who generated the random allocation sequence, who enrolled participants, and who assigned participants to the sequence of interventions	The random allocation sequence was jointly developed by the research team; participants were recruited by researchers through local educational institutions and communities (research information was released in the form of internal notices, and parents or legal guardians voluntarily registered their children to participate after fully understanding the research content); the intervention sequence was assigned by experimental implementers in accordance with the

			preset order.
Blinding	11a	If done, who was blinded after assignment to interventions (for example, participants, care providers, those assessing outcomes) and how	Due to the significant difference in interaction modes between the two interventions (the experimental group included gamified feedback, while the control group did not), blinding could not be implemented for participants or experimental implementers. However, blinding was applied during the data collection and analysis phases: researchers responsible for data entry and statistical analysis were unaware of the participants' intervention sequences and only processed data using anonymized identifiers to reduce measurement and analysis bias. All participant data were anonymized and de-identified to avoid associating any personal identity information.
Similarity of interventions	11b	If relevant, description of the similarity of interventions	Both groups used identical inhaler hardware with consistent core

			operations and structures. Only the experimental group incorporated a gamified feedback mechanism (BreatheBuddy system), while the control group did not. The appearance of both inhalers was moderately optimized for fun to improve children’s acceptance, and both used disposable environmental protection straws as mouthpieces to avoid cross-contamination.
Statistical methods	12a	Statistical methods used to compare groups for primary and secondary outcomes which are appropriate for crossover design (that is, based on within participant comparison)	The Shapiro-Wilk test was used to detect the normality of the data. Paired samples t-tests were used to analyze differences between the two intervention conditions for normally distributed data; if data did not meet the assumptions of normality, the Mann–Whitney U test was used as a nonparametric alternative. All statistical analyses were based on within-participant comparisons,

			accounting for the correlation of participants' own data. The scores of PENS, GUESS, and SUS were standardized to the range of 1 to 100 before statistical analysis.
Additional analyses	12b	Methods for additional analyses, such as subgroup analyses and adjusted analyses	No subgroup or adjusted analyses were conducted. Thematic analysis was used to analyze qualitative interview data (after transcription) to supplement and interpret the quantitative results. The thematic analysis process included familiarizing with data, initial open coding, aggregating similar codes to form themes, reviewing and revising themes, naming and defining themes, and integrating to form explanatory narratives. Two researchers independently coded to ensure the rigor of the analysis.
**Results**<br>Participant flow (a diagram is strongly	13a	The numbers of participants who were randomly	A total of 20 participants were randomly assigned

recommended)		assigned, received intended treatment, and were analysed for the primary outcome, separately for each sequence and period (a diagram is strongly recommended)	(10 in each intervention sequence). All participants completed both preset intervention treatments with no dropouts, exclusions, or loss to follow-up, and all were included in the primary outcome analysis. The participant flow is illustrated in the participant flow diagram (Figure 8), which presents the flow process separately for each intervention sequence (experimental group → control group, control group → experimental group).
Losses and exclusions	13b	No of participants excluded at each stage, with reasons, separately for each sequence and period	No participants were excluded at any stage of the study, and all 20 participants completed the entire experimental process (including two intervention sessions, questionnaire filling, and semi-structured interviews).
Recruitment	14a	Dates defining the periods of	Specific dates for recruitment and

		recruitment and follow-up	follow-up were not recorded. Participants were recruited through local educational institutions and communities, and the experiment was conducted immediately after recruitment was completed; no follow-up phase was included in this study.
Trial end	14b	Why the trial ended or was stopped	The trial was completed as planned and ended because all 20 participants finished the experiment (including intervention, questionnaire, and interview), the preset sample size was achieved, and no early termination was required.
Baseline data	15	A table showing baseline demographic and clinical characteristics by sequence and period	Among the 20 participants, there were 10 boys and 10 girls, with a mean age of 6.9 years (SD = 0.79). All participants had prior experience in inhaler use, with balanced baseline characteristics and no significant between-group differences.

			(Baseline characteristics are not presented separately by sequence because all participants received both interventions and had consistent baseline profiles.)
Numbers analysed	16	Number of participants (denominator) included in each analysis and whether the analysis was by original assigned groups	All 20 participants were included in each analysis (quantitative data analysis and qualitative interview analysis). Analyses were conducted based on the two intervention conditions (experimental group and control group) rather than the original allocation sequence, which is consistent with the requirements for crossover design analyses.
Outcomes and estimation	17a	For each primary and secondary outcome, results including estimated effect size and its precision (such as 95% confidence interval) should be based on within participant comparisons. In addition, results for each intervention in	① Primary outcomes: Compared with the control group, the experimental group demonstrated significantly improved inhalation accuracy, longer breath-holding duration (experimental group: mean 12.20s,

		each period are recommended	SD=2.11, 95% CI 11.263s-13.131s; control group: mean 7.92s, SD=2.27, 95% CI 6.909s-8.921s), and more stable breathing patterns (smaller variance and standard deviation of RESP data, $p < 0.001$). The mean respiratory rate of the experimental group was 9.97rpm (SD=0.57, 95% CI 9.719 rpm -10.229 rpm), significantly lower than that of the control group (10.47rpm, SD=0.82, 95% CI 10.110rpm - 10.837rpm, p=.032). ② Secondary outcomes: The experimental group had significantly higher PENS scores (SS = 93.83, 95% CI 92.819-94.847), GUESS scores (median = 87.92, 95% CI 86.196-88.291), and SUS scores (88.96, 95% CI 86.394-91.522) than the control group ($p < 0.001$), along with reduced anxiety and increased attention concentration. All results were based on within-

			participant comparisons, and results for each intervention in each period are not reported separately.
Binary outcomes	17b	For binary outcomes, presentation of both absolute and relative effect sizes is recommended	Not applicable. All outcome measures in this study were continuous variables (such as breath-holding duration, scale scores, respiratory rate), and no binary outcomes were assessed.
Ancillary analyses	18	Results of any other analyses performed, including subgroup analyses and adjusted analyses, distinguishing prespecified from exploratory	No subgroup or adjusted analyses were conducted. Only thematic analysis of qualitative interview data was performed, which was a prespecified supplementary analysis to interpret the quantitative results. Five core themes were extracted from the interview data: (1) intrinsic motivation and behavior change, (2) flow experience and participation, (3) game mechanism and playability, (4) feedback mechanism and breathing accuracy,

			(5) caregiver and expert feedback.
Harms	19	Describe all important harms or untended effects in a way that accounts for the design (for specific guidance, see CONSORT for harms)	This study involved simulated inhalation training without the use of actual medications. The inhaler used a disposable environmental protection straw as the mouthpiece to avoid cross-contamination. No adverse events or unintended effects were observed in any participants during the experiment, and no feedback regarding discomfort was received from participants, caregivers, or experts.
**Discussion**<br>Limitations	20	Trial limitations, addressing sources of potential bias, imprecision, and if relevant, multiplicity of analyses. Consider potential carry over effects	Limitations of this study include a small sample size (20 participants) with a relatively concentrated age range (6-8 years), which may limit the generalizability of the research results; lack of long-term follow-up, making it impossible to evaluate the impact of the system on compliance and efficacy stability in

			long-term use; potential risk that some users may excessively pursue physiological indicators (such as excessively long breath-holding) and ignore the comfort and safety of the breathing process. Potential biases primarily include the inability to blind participants and experimental implementers; however, this bias was mitigated by applying blinding during the data collection and analysis phases. A 10-minute washout period was implemented to reduce the impact of carry-over effects. No multiple analyses were conducted, thus avoiding issues related to multiplicity.
Generalisability	21	Generalisability (external validity, applicability) of the trial findings	The findings of this study are generalizable to children with asthma aged 6–8 years who have prior experience in inhaler use and can provide a reference for clinical inhaler

			training in this population. A limitation is the small sample size and limited recruitment scope (local communities and educational institutions). Future studies should expand the sample size and recruitment scope, and verify the system effect in a larger sample and real clinical environment to enhance the generalisability of the results.
Interpretation	22	Interpretation consistent with results, balancing benefits and harms, and considering other relevant evidence	The interpretation of results is consistent with the study findings, balances benefits and harms, and considers other relevant evidence. The results demonstrate that the BreatheBuddy system significantly improves children's inhalation skills and treatment compliance, reduces treatment-related anxiety, and is free of adverse events. Compared with existing studies that mostly rely on non-gamified training methods or use games as additional

			distraction tools, this study embeds breathing behaviors into the game interaction mechanism, addressing the limitations of current gamified interventions and providing a new tool and approach for inhaler training in children with asthma. These findings are consistent with relevant research conclusions (such as the role of real-time feedback in shaping healthy behaviors) and offer novel insights. Future research can further explore personalized adaptation mechanisms and their application potential in a wider range of clinical scenarios.
**Other information**<br>Registration	23	Registration number and name of trial registry	This trial was not registered, so no registration number or registry name is available.
Protocol	24	Where the full trial protocol can be accessed, if available	The full trial protocol was not made publicly available, so no access information can be provided.

Funding	25	Sources of funding and other support (such as supply of drugs), role of funders	This study was funded by the Humanities and Social Science Fund of the Ministry of Education of China, grant number 24YJAZH070. The funders were not involved in study design, data collection, analysis, interpretation of results, or manuscript preparation. This study has been reviewed and approved by the Ethics Committee of Hubei University of Technology (approval number: HBUT20250043), in line with the relevant norms of human research ethics. No cash compensation was provided; each child was given a little yellow duck-themed card as a souvenir after the experiment.
