# Supplementary material for: Gamified Feedback-Based Training System for Pediatric Asthma Inhaler Use: Mixed Methods Randomized Crossover Study
Source: JMIR Serious Games. 2026 May 4;14:e85673. doi: 10.2196/85673 (PMC13138708; doi:10.2196/85673)
Supplement: Checklist 2 [file games-v14-e85673-s003.pdf]

Section/Topic	Item No.	CONSORT Checklist Item	EHEALTH Extensions (additions to, or clarification of the CONSORT item)	*Importance*
**TITLE & ABSTRACT**	1a	Identification as a randomized trial in the title	i) Mode of delivery identified: serious game/computer-based gamified feedback system (integrating physical inhaler + interactive software); ambiguous terms avoided.<br>ii) Non-web-based components mentioned: physical inhaler with Arduino-based airflow sensor, researcher-assisted training.<br>iii) Primary target group mentioned: children with pediatric asthma	*Essential*<br>*Highly Recommended*<br>*Essential*
	1b	Structured summary of trial design, methods, results, and conclusions; NPT extension: Description of experimental treatment, comparator, care providers, centers, and blinding status	**Methods**:<br>i) Key intervention features: BreatheBuddy (MDA game design framework/SDT, real-time breathing feedback via "little yellow duck diving" narrative, airflow sensor + TouchDesigner interactive layer); comparator: standard inhaler training (no gamified feedback). Validated theories (MDA, Self-Determination Theory) noted.<br>ii) Human involvement: fully automated gamified feedback, 1-2 researchers assisted with training/questionnaire guidance (no healthcare provider co-intervention).<br>iii) Recruitment/assessment: Recruited offline (local community health centers/kindergartens); assessments: objective respiratory data (RESP/airflow sensor) + self-assessed online questionnaires (PENS/GUESS/SUS via Questionnaire Star); purely laboratory-based trial with no face-to-face clinical components.<br>iv) Results: 20 participants (10 boys/10 girls) all analysed; use data reported (3-min training per intervention, real-time breathing data monitoring, adherence via PENS scores).<br>v) Negative trials: Not applicable (trial positive for primary/secondary outcomes).	*Essential*<br>*Highly Recommended*<br>*Highly Recommended*<br>*Highly Recommended*
**INTRODUCTION** Background	2a	Scientific background and explanation of	i) Problem/solution: Pediatric asthma inhaler use errors/low adherence; BreatheBuddy as a **stand-alone gamified training intervention** for 6-8-	*Essential*

Section/Topic	Item No.	CONSORT Checklist Item	EHEALTH Extensions (additions to, or clarification of the CONSORT item)	*Importance*
and objectives		rationale	year-old asthmatic children, designed to replace traditional non-interactive inhaler training.<br>ii) Scientific background: High inhaler error rates in children (8%-22% correct use); existing eHealth/gamified interventions lack integration of inhaler as input device; BreatheBuddy combines real-time breathing feedback and gamified design. Comparator (standard training) justified as clinical gold standard for inhaler education.	
	2b	Specific objectives or hypotheses	No EHEALTH-specific additions; study questions (RQ1-RQ3) and hypotheses (H1-H4) clearly stated for inhaler skill, adherence, and user experience outcomes.	*Essential*
**METHODS -** Trial design	3a	Description of trial design (such as parallel, factorial) including allocation ratio	No EHEALTH-specific additions; single-factor repeated-measures randomized crossover design, allocation ratio 1:1 (all participants received both interventions).	*Essential*
	3b	Important changes to methods after trial commencement (such as eligibility criteria), with reasons	i) No major bug fixes, system downtimes, content changes, or unexpected events (e.g., staff/system failures) during the trial; BreatheBuddy hardware/software frozen for the study period.	*Highly Recommended*
Participants	4a	Eligibility criteria for participants	i) Computer/Internet literacy: no independent literacy required (researchers provided full operation guidance for the gamified system).<br>ii) Recruitment/assessment: Recruited offline (local community health centers/kindergartens, parental voluntary registration); quasi-anonymous data processing (no personal identifiers linked to results); technical measures (disposable mouthpieces) for hygiene, no multiple identity issues.<br>iii) Informed consent: Written consent from parents/legal guardians, verbal consent from	*Highly Recommended*<br>*Highly Recommended*<br>*Essential*

Section/Topic	Item No.	CONSORT Checklist Item	EHEALTH Extensions (additions to, or clarification of the CONSORT item)	*Importance*
			children; study purpose/process/risk clearly explained; no consent documentation published as appendix (available in research laboratory).	
	4b	Settings and locations where the data were collected	i) Outcome assessment: objective respiratory data (RESP/airflow sensor) + self-assessed online questionnaires (PENS/GUESS/SUS).<br>ii) Institutional affiliation: Hubei University of Technology affiliation clearly presented in recruitment materials; no bias from prestigious affiliation reported.	*Essential*<br>*Recommended*
Interventions	5	The interventions for each group with sufficient details to allow replication, including how and when they were actually administered	i) Developers/sponsors: Research team from Hubei University of Technology (supervised by doctoral advisors); no external owners/sponsors; conflict of interest stated (team = system developers).<br>ii) Development process: Preliminary questionnaire/semi-structured interviews (children/parents/2 pediatric nurses); multiple workshop discussions; prototype testing/calibration (hardware/software).<br>iii) Revisions/updating: BreatheBuddy frozen during trial (no major revisions); hardware (3D-printed inhaler v1.0), software (TouchDesigner game v1.0) version numbers noted.<br>iv) Quality assurance: Pre-trial device calibration; standardized data collection protocols; validated scales (PENS/GUESS/SUS) for assessment.<br>v) Replicability: Device specifications/screenshots provided, Arduino/TouchDesigner source code available; flowchart of breathing data feedback algorithm included.<br>vi) Digital preservation: Hardware/software prototype archived in the research laboratory; demo version of the game available for peer review (no public URL, laboratory-based access).<br>vii) Access: Free for participants, laboratory-based use (Wi-Fi for data transmission); no	*Highly Recommended*<br>*Highly Recommended*<br>*Highly Recommended*<br>*Highly Recommended*<br>*Highly Recommended*<br>*Essential*<br>*Essential*<br>*Essential*<br>*Highly Recommended*<br>*Essential*<br>*Highly Recommended*<br>*Essential*

**Section/Topic**	**Item No.**	**CONSORT Checklist Item**	**EHEALTH Extensions (additions to, or clarification of the CONSORT item)**	***Importance***
			membership/fee; disposable mouthpieces for hygiene; backdoor/demo mode for reviewers available.<br>viii) Mode/features/theory: MDA game design framework + SDT; core features: real-time inhalation/hold/exhalation feedback (yellow duck narrative), physical (Arduino/airflow sensor) + visual/audio (TouchDesigner) layers; comparator: standard inhaler training (no gamified feedback, minimal researcher guidance). Content developed by the research team with pediatric nurse input; tailored to children's cognitive level (6-8 years) with progress tracking/real-time feedback; asynchronous researcher assistance.<br>ix) Use parameters: Intended dose: 3-minute structured training session per intervention; ad libitum practice during sessions; no additional timing/frequency recommendations.<br>x) Human involvement: 1-2 researchers (child communication experience) for training/questionnaire guidance; trial-specific involvement higher than routine use (no researcher assistance in clinical practice); no healthcare provider co-intervention.<br>xi) Prompts/reminders: Intrinsic in-game visual/audio feedback (no external prompts/reminders); no trial-specific prompts beyond routine use.<br>xii) Co-interventions: Researcher-provided training guidance/questionnaire explanation; no additional training/support beyond trial protocol.	
Outcomes	6a	Completely defined pre-specified primary and secondary outcome measures, including how and when they were assessed	i) Online questionnaires: PENS/GUESS/SUS are validated scales adapted for online use; CHERRIES items applied for Questionnaire Star deployment.<br>ii) Use measurement: Real-time monitoring via airflow/RESP sensor (logins, session duration, inhalation depth, breath-hold time); adherence (PENS), engagement (GUESS) as process	*Essential*<br>*Highly Recommended*<br>*Highly Recommended*

Section/Topic	Item No.	CONSORT Checklist Item	EHEALTH Extensions (additions to, or clarification of the CONSORT item)	*Importance*
			outcomes; "session" defined as 3-minute structured training (no idle timeout).<br>iii) Qualitative feedback: Semi-structured interviews (participants/parents/healthcare providers) post-intervention; thematic analysis of feedback on usability/breathing accuracy.	
	6b	Any changes to trial outcomes after the trial commenced, with reasons	No EHEALTH-specific additions; no outcome changes during the trial.	*Essential*
Sample size	7a	How sample size was determined NPT: When applicable, details of whether and how the clustering by care provides or centers was addressed	i) Sample size based on pilot study effect estimates, child participant recruitment constraints, and research resources; expected attrition pre-estimated as 0 (no loss anticipated for laboratory-based trial).	*Highly Recommended*
	7b	When applicable, explanation of any interim analyses and stopping guidelines	No EHEALTH-specific additions; no interim analyses/stopping guidelines (small sample, pre-specified sample size of 20).	*Essential*
Randomisation: Sequence generation	8a	Method used to generate the random allocation sequence NPT: When applicable, how care providers were allocated to each trial group	No EHEALTH-specific additions; sequence based on participant arrival order (first 10: BreatheBuddy → control; last 10: control → BreatheBuddy).	*Essential*
	8b	Type of randomisation; details of any restriction (such as blocking and block	No EHEALTH-specific additions; unrestricted randomisation, block size = 10.	*Essential*

Section/Topic	Item No.	CONSORT Checklist Item	EHEALTH Extensions (additions to, or clarification of the CONSORT item)	*Importance*
		size)		
Allocation concealment mechanism	9	Mechanism used to implement the random allocation sequence, describing any steps taken to conceal the sequence until interventions were assigned	No EHEALTH-specific additions; no allocation concealment (crossover design, arrival-based sequence).	*Essential*
Implementation	10	Who generated the random allocation sequence, who enrolled participants, and who assigned participants to interventions	No EHEALTH-specific additions; research team generated the sequence, enrolled participants, and assigned interventions.	*Essential*
Blinding	11a	If done, who was blinded after assignment to interventions (for example, participants, care providers, those assessing outcomes) and how NPT: Whether or not administering co-interventions were blinded to group assignment	i) Blinding status: Participants/researchers administering interventions unblinded (gamified feedback vs. standard training visually distinct); data analysts/collectors blinded (anonymous participant IDs, no knowledge of intervention sequence). Acknowledged that blinding participants is impossible in gamified eHealth trials.<br>ii) Informed consent bias: No bias (participants/parents informed of both interventions, no "intervention of interest" highlighted).	*Essential*<br>*Highly Recommended*
	11b	If relevant, description of the similarity of interventions	No EHEALTH-specific additions; interventions share the same inhaler hardware, but BreatheBuddy has an additional gamified feedback module (no similarity for the core	-

Section/Topic	Item No.	CONSORT Checklist Item	EHEALTH Extensions (additions to, or clarification of the CONSORT item)	*Importance*
			intervention).	
Statistical methods	12a	Statistical methods used to compare groups for primary and secondary outcomes NPT: When applicable, details of whether and how the clustering by care providers or centers was addressed	i) Attrition/missing values: No missing data/attrition (20 participants completed all interventions); no imputation techniques needed (complete case analysis justified only for zero attrition).	*Highly Recommended*
	12b	Methods for additional analyses, such as subgroup analyses and adjusted analyses	No EHEALTH-specific additions; thematic analysis for qualitative interview data; no quantitative subgroup analyses (homogeneous sample: 6-8-year-olds).	*Essential*
Ethics & Informed Consent	X26	*(Not a CONSORT item)*	i) Ethics committee approval: HBUT20250043, Ethics Committee of Hubei University of Technology.<br>ii) Informed consent: Offline written consent (parents/guardians), verbal consent (children); clear information on study purpose/risks/voluntary withdrawal.<br>iii) Safety/privacy: Anonymous/de-identified data processing; disposable mouthpieces (infection control); no personal information in published materials; strict data confidentiality; no hotline needed (laboratory-based trial with real-time researcher supervision).	*Highly Recommended*<br>*Highly Recommended*<br>*Highly Recommended*
**RESULTS** - Participant flow	13a	For each group, the numbers of participants who were randomly assigned, received intended treatment,	No EHEALTH-specific additions; 20 participants assigned to both interventions, all received intended treatment, all analysed for primary outcomes (crossover design).	*Essential*

Section/Topic	Item No.	CONSORT Checklist Item	EHEALTH Extensions (additions to, or clarification of the CONSORT item)	*Importance*
		and were analysed for the primary outcome NPT: The number of care providers or centers performing the intervention in each group and the number of patients treated by each care provider in each center		
	13b	For each group, losses and exclusions after randomisation, together with reasons	i) Attrition/use visualization: No losses/exclusions; participant flowchart provided; breathing use data (breath-hold time/respiratory frequency) presented as statistical charts (no survival curve needed for zero attrition).	*Highly Recommended*
Recruitment	14a	Dates defining the periods of recruitment and follow-up	i) No critical secular events (e.g., Internet/hardware changes) during the study period; specific recruitment dates not reported.	*Highly Recommended*
	14b	Why the trial ended or was stopped [early]	No EHEALTH-specific additions; trial ended after completing the pre-specified sample size (20 participants); no early termination.	-
Baseline data	15	A table showing baseline demographic and clinical characteristics for each group NPT: When applicable, a description of care providers (case volume, qualification,	i) eHealth-relevant demographics: 20 participants (10 boys/10 girls), mean age 6.9y (SD=0.79), all with inhaler use experience; computer/Internet literacy not reported (researcher-assisted use); no digital divide issues noted.	*Essential*

Section/Topic	Item No.	CONSORT Checklist Item	EHEALTH Extensions (additions to, or clarification of the CONSORT item)	*Importance*
		expertise, etc.) and centers (volume) in each group		
Numbers analysed	16	For each group, number of participants included in each analysis and whether the analysis was by original assigned groups	i) Denominators/use definition: 20 participants per group; "use" defined as active inhaler operation with sensor-recorded breathing data; no additional use thresholds (all participants used the device as intended).<br>ii) Analysis: Intent-to-treat (ITT) analysis; no secondary "user-only" analysis (all participants were valid users).	*Highly Recommended Essential*
Outcomes and estimation	17a	For each primary and secondary outcome, results for each group, and the estimated effect size and its precision (such as 95% confidence interval)	i) Process outcomes: Use metrics reported (3-min session duration, sensor-recorded inhalation depth/breath-hold time, respiratory frequency); "session" defined as structured training with continuous data collection (no idle timeout); effect sizes (mean/median ± SD, 95% CI, p-values) reported for all use/clinical outcomes.	*Highly Recommended*
	17b	For binary outcomes, presentation of both absolute and relative effect sizes is recommended	No EHEALTH-specific additions; no binary outcomes (all outcomes continuous/ordinal).	-
Ancillary analyses	18	Results of any other analyses performed, including subgroup analyses and adjusted analyses, distinguishing pre-specified from exploratory	i) Subgroup analysis: No user-only subgroup analysis (all participants used the intervention); qualitative thematic analysis of interview data (exploratory) reported to complement quantitative results.	*Highly Recommended*
Harms	19	All important harms or unintended	i) Harms/technical issues: No physical harm, privacy breaches, or technical failures during the	*Highly Recommended*

**Section/Topic**	**Item No.**	**CONSORT Checklist Item**	**EHEALTH Extensions (additions to, or clarification of the CONSORT item)**	***Importance***
		effects in each group	trial.<br>ii) Qualitative feedback: Unintended feedback (children: partial low task difficulty/monotony with repetition; parents: no negative feedback; experts: need for progressive difficulty); no unintended positive/negative effects on asthma symptoms.	*Highly Recommended*
Interpretation/ Principal Findings	22	Interpretation consistent with results, balancing benefits and harms, and considering other relevant evidence NPT: In addition, take into account the choice of the comparator, lack of or partial blinding, and unequal expertise of care providers or centers in each group	i) Interpretation: Study questions/hypotheses answered with primary (breathing accuracy) and process (use/adherence) outcomes; BreatheBuddy significantly outperformed standard training.<br>ii) Future research: Unanswered questions (personalization, long-term follow-up, larger samples, clinical asthma outcomes); future research recommended for broad clinical application and age range expansion.	*Essential*<br>*Highly Recommended*
**DISCUSSIO N** - Limitations	20	Trial limitations, addressing sources of potential bias, imprecision, and, if relevant, multiplicity of analyses	i) eHealth-specific limitations: No participant blinding (inherent to gamified trials); small sample/ narrow age range (6-8y) (limited generalizability); no multiple outcomes/I type error risk; no use/usability bias or unexpected events. Additional limitations: short-term intervention (no long-term follow-up), no personalization of gamified feedback.	*Essential*
Generalisability	21	Generalisability (external validity, applicability) of the trial findings NPT: External validity of the trial findings	i) Population generalizability: Limited to 6-8-year-old asthmatic children from local Chinese communities; need validation for younger/older children, different regions/cultures, and general Internet/clinical populations.<br>ii) RCT vs. routine use: Trial-specific researcher	*Highly Recommended*<br>*Highly Recommended*

Section/Topic	Item No.	CONSORT Checklist Item	EHEALTH Extensions (additions to, or clarification of the CONSORT item)	*Importance*
		according to the intervention, comparators, patients, and care providers or centers involved in the trial	assistance higher than routine clinical/family use (no researcher support in routine application); no external prompts/reminders in trial (parents can provide guidance in routine use); BreatheBuddy adaptable to routine clinical training (no laboratory-only equipment).	
**OTHER INFORMATION** - Registration	23	Registration number and name of trial registry	No EHEALTH-specific additions; trial registration not reported.	*Essential*
Protocol	24	Where the full trial protocol can be accessed, if available	No EHEALTH-specific additions; full protocol available in the research laboratory (no public access).	-
Funding	25	Sources of funding and other support, role of funders	No EHEALTH-specific additions; funded by Humanities and Social Science Fund of the Ministry of Education of China (24YJAZH070); funders had no role in study design/analysis/reporting.	*Essential*
Competing interests	X27	(Not a CONSORT item)	i) Competing interests: Research team = developers of BreatheBuddy; no other financial/non-financial competing interests; clear statement of study team-system relationship.	*Highly Recommended*
